# Genome Sequence of the Pea Aphid *Acyrthosiphon pisum*

**DOI:** 10.1371/journal.pbio.1000313

**Published:** 2010-02-23

**Authors:** The International Aphid Genomics Consortium

**Affiliations:** University of California Davis, United States of America

## Abstract

Aphids are important agricultural pests and also biological models for studies of insect-plant interactions, symbiosis, virus vectoring, and the developmental causes of extreme phenotypic plasticity. Here we present the 464 Mb draft genome assembly of the pea aphid *Acyrthosiphon pisum*. This first published whole genome sequence of a basal hemimetabolous insect provides an outgroup to the multiple published genomes of holometabolous insects. Pea aphids are host-plant specialists, they can reproduce both sexually and asexually, and they have coevolved with an obligate bacterial symbiont. Here we highlight findings from whole genome analysis that may be related to these unusual biological features. These findings include discovery of extensive gene duplication in more than 2000 gene families as well as loss of evolutionarily conserved genes. Gene family expansions relative to other published genomes include genes involved in chromatin modification, miRNA synthesis, and sugar transport. Gene losses include genes central to the IMD immune pathway, selenoprotein utilization, purine salvage, and the entire urea cycle. The pea aphid genome reveals that only a limited number of genes have been acquired from bacteria; thus the reduced gene count of *Buchnera* does not reflect gene transfer to the host genome. The inventory of metabolic genes in the pea aphid genome suggests that there is extensive metabolite exchange between the aphid and *Buchnera*, including sharing of amino acid biosynthesis between the aphid and *Buchnera*. The pea aphid genome provides a foundation for post-genomic studies of fundamental biological questions and applied agricultural problems.

## Abstract 2

The genome of the pea aphid shows remarkable levels of gene duplication and equally remarkable gene absences that shed light on aspects of aphid biology, most especially its symbiosis with *Buchnera*.

## Author Summary

Aphids are common pests of crops and ornamental plants. Facilitated by their ancient association with intracellular symbiotic bacteria that synthesize essential amino acids, aphids feed on phloem (sap). Exploitation of a diversity of long-lived woody and short-lived herbaceous hosts by many aphid species is a result of specializations that allow aphids to discover and exploit suitable host plants. Such specializations include production by a single genotype of multiple alternative phenotypes including asexual, sexual, winged, and unwinged forms. We have generated a draft genome sequence of the pea aphid, an aphid that is a model for the study of symbiosis, development, and host plant specialization. Some of the many highlights of our genome analysis include an expanded total gene set with remarkable levels of gene duplication, as well as aphid-lineage-specific gene losses. We find that the pea aphid genome contains all genes required for epigenetic regulation by methylation, that genes encoding the synthesis of a number of essential amino acids are distributed between the genomes of the pea aphid and its symbiont, *Buchnera aphidicola,* and that many genes encoding immune system components are absent. These genome data will form the basis for future aphid research and have already underpinned a variety of genome-wide approaches to understanding aphid biology.

## Introduction

**Figure 1:**
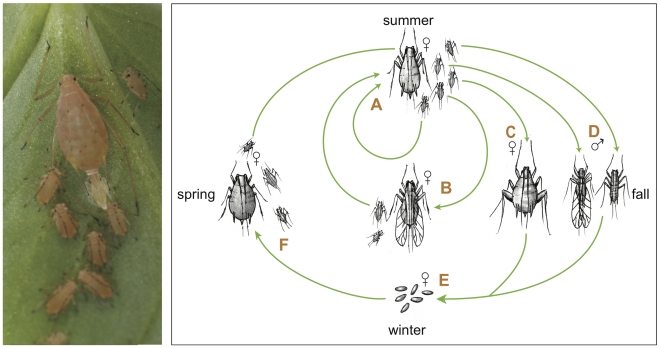
The pea aphid life cycle. During the spring and summer months, asexual females give birth to live clonal offspring (see photo). These offspring undergo four molts during larval development to become (A) unwinged or (B) winged asexually reproducing adults. Winged individuals, capable of dispersing to new plants, are induced by crowding or stress during prenatal stages. After repeated cycles of asexual reproduction, shorter autumn day lengths trigger the production of (C) unwinged sexual females and (D) males, which can be winged or unwinged in pea aphids, depending on genotype. After mating, oviparous sexual females deposit (E) overwintering eggs, which hatch in the spring to produce (F) wingless, asexual females. In some populations, especially in locations without a cold winter, the sexual and egg-producing portions of the life cycle are eliminated, leading to continuous cycles of asexual reproduction (photo by N. Gerardo; illustration by N. Lowe).

Aphids are small, soft-bodied insects with elaborate life cycles that include all-female, parthenogenetic generations that alternate with sexual generations (Figure 1). Aphids feed exclusively on plant phloem sap by inserting their slender mouthparts into sieve elements, the primary food conduits of plants. Many of the ∼5,000 aphid species attack agricultural plants and inflict damage both through the direct effects of feeding and by vectoring debilitating plant viruses. Annual worldwide crop losses due to aphids are estimated at hundreds of millions of dollars [1],[2],[3].

**Figure 2:**
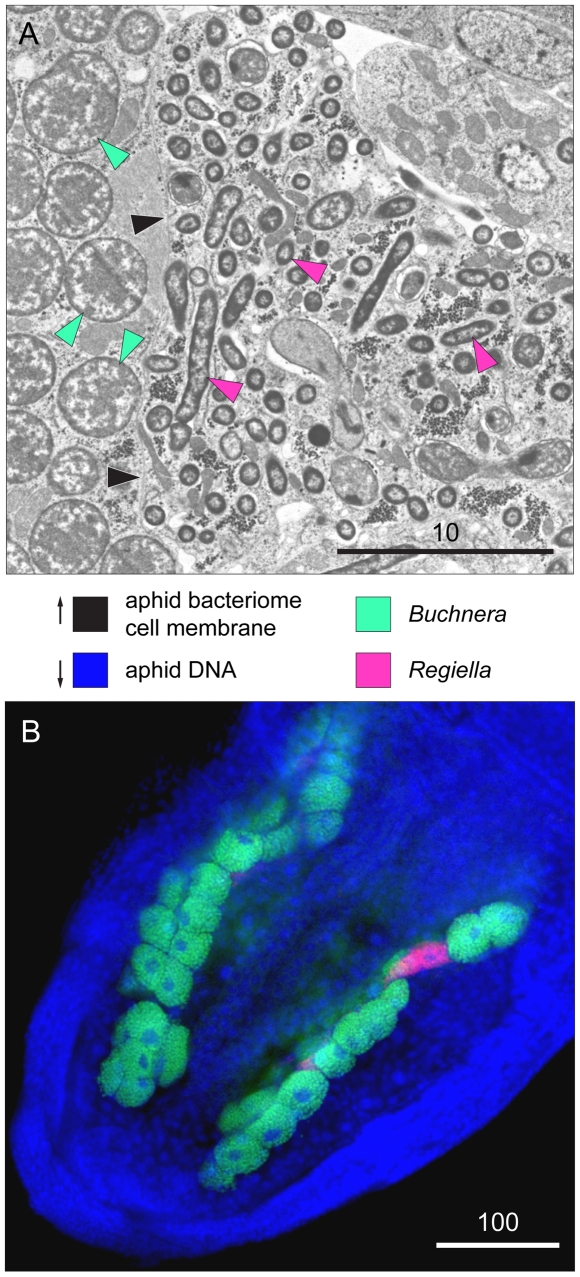
*Buchnera aphidicola* and *Regiella insecticola* within a pea aphid embryo. (A) Transmission electron micrograph showing elongate *Regiella* cells within a bacteriocyte (pink arrows) and nearby bacteriocytes containing *Buchnera* (green arrows). Black arrows indicate the bacteriome cell membrane (photo by J. White and N. Moran). Scales are in microns. (B) Position of symbiont-containing bacteriocytes within the abdomen as revealed by fluorescent *in situ* hybridization using diagnostic probes. Blue is a general DNA stain, highlighting aphid nuclei, red indicates *Regiella*, and green indicates *Buchnera* (photo by R. Koga).

Phloem sap is rich in simple sugars but contains an unbalanced mixture of amino acids. This unbalanced diet is compensated for by the intracellular mutualistic bacterium, *Buchnera aphidicola* (Figure 2), which has coevolved with aphids [4] and provides essential amino acids that are absent or rare in phloem sap [5]. Additionally, some aphids, including the pea aphid, have facultative associations with a variety of other heritable bacterial symbionts that provide ecological benefits, such as heat tolerance and resistance to parasitoids [6].

Aphids, which are essentially plant parasites, have evolved complex life cycles involving extensive phenotypic plasticity [1]. They produce individuals with multiple distinct phenotypes (polyphenism), so that individuals with identical genotypes can develop into one of several alternative phenotypes, each adapted to a particular ecological situation (Figure 1). Aphids develop as asexual live-bearing females or as sexual males and egg-laying females during different seasons. Asexual females occur as sedentary wingless forms or as winged forms specialized for dispersal. In many aphid species, individuals from different stages of the life cycle may feed on distinct sets of plant species. In addition, some aphid species produce morphs that are specialized to resist desiccation or to defend the colony. Asexual forms have evolved a highly modified meiosis that omits the reduction division of Meiosis I, allowing apomictic parthenogenesis. Parthenogenetically produced embryos develop directly within their mothers, sometimes before the birth of the mother herself, so that females can end up carrying both their daughters and their granddaughters within them. This telescoping of generations promotes short generation times, allowing aphid colonies to rapidly exploit new resources. Like other hemimetabolous insects, aphids undergo an incomplete metamorphosis from juvenile to adult stages.

Here we present the genome sequence of the pea aphid, *Acyrthosiphon pisum*. This aphid, which is widely used in laboratory studies, attacks legume crops (Fabaceae) and is closely related to important crop pests, including the green peach aphid (*Myzus persicae*) and the Russian wheat aphid (*Diuraphis noxia*) [7]. This first published hemimetabolous genome, coupled with the genomes of its obligate and facultative bacterial symbionts [8],[9],[10], provides a strong foundation for exploring the genetic basis of coevolved symbiotic associations, of host plant specialization, of insect-plant interactions, and of the developmental causes of extreme phenotypic plasticity. We first provide an overview of the general features of the pea aphid genome and then review findings of manual gene annotation efforts focused on genes related to symbiosis, insect-plant interactions, and development. Additional findings from these annotation projects can be found in multiple companion papers [8],[11]–[39].

## Results and Discussion

### General Features of the Pea Aphid Genome

#### Genome sequence and organization

The haploid pea aphid genome of four holocentric chromosomes (three autosomes and one X chromosome) was estimated by flow cytometry for the sequenced pea aphid line LSR1.AC.G1 to be 517 Mb (*SE* = 3.15 Mbp, *N* = 7). Sanger sequencing of DNA samples from line LSR1.AC.G1 produced 4.4 million raw sequence reads (6.2× genome coverage, Table S1) of which 3.05 million were in the final assembly. This Acyr 1.0 assembly contains 72,844 contigs, with an N50 length of 10.8 kb and a total length of 446.6 Mb. The scaffold N50 is 88.5 kb, and scaffolds including gaps between the ordered and oriented contigs had a total length of 464 Mb. To estimate the gene coverage of the assembly, 97,878 ESTs (5′-EST: 49,991; 3′-EST: 47,837; [33]) generated from a full-length *A. pisum* cDNA library were mapped to the Acyr 1.0 assembly. Ninety-nine percent of these EST sequences were mapped in Acyr 1.0, and 81% of the clones had both 5′- and 3′-ESTs mapping to the same scaffold with appropriate separation distance and opposite orientations. No sequences with high similarity to the ∼170,000 available ESTs were found in the unassembled reads, suggesting that few protein-coding genes remain in the unassembled fraction of the dataset.

#### GC content

The assembled regions of the pea aphid genome have the lowest GC content of any insect genome sequenced to date; at 29.6%, pea aphid GC content is 5.2% lower than that of *Apis mellifera* at 34.8% [40]. Computed over all concatenated transcripts pea aphid GC content averages 38.8% (*SD* = 8.4, *N* = 37,994), a value similar to that of *Apis mellifera* (mean = 38.6%, *SD* = 9.7, *N* = 17,182) (Table S2).

#### Gene model prediction

Prior to this project, less than 200 pea aphid genes had been sequenced. Thus, we performed automated gene predictions to aid study of the pea aphid gene repertoire. High-quality gene models with either partial or full-length EST and/or protein homology support computed by NCBI's gene prediction pipeline serve as a core set of 10,249 protein-coding gene models and are integrated into the public RefSeq databases at NCBI. Since the number of gene models with EST or protein homology support is expected to be smaller than the true number of protein-coding genes in the pea aphid genome, additional gene models were calculated using six additional gene prediction programs and combined, using GLEAN [41], into a consensus set of 24,355 additional gene models (Table 1). When compared to 2,089 exons of known origin and sequence, the GLEAN consensus gene models contained the highest number of bases overlapping the known exons. Other details of this comparison are in Table S3, and a comparison of pea aphid and other arthropod gene structures is shown in Table S4.

**Table 1:** Summary of pea aphid gene model sets.

Gene Modeling Software	Prediction Type	Gene Models	mRNAs	Number of Exons Per mRNA	Average mRNA Length	Average Exon Length	Total Number of Exons	Total Exon Length
NCBI RefSeq	Evidence	11,089	11,308	7.6	1,908 bp	251 bp	86,018	21.6 Mb
NCBI Gnomon	*ab initio*	37,994	37,994	3.9	887 bp	222 bp	149,183	33.3 Mb
Augustus	*ab initio* plus evidence	33,713	40,594	5.3	982 bp	223 bp	147,909	33.1 Mb
Fgenesh	*ab initio*	30,846	30,846	4.5	1,048 bp	232 bp	139,357	32.3 Mb
Fgenesh++	*ab initio* plus evidence	26,773	26,773	4.9	1,148 bp	236 bp	130,509	30.7 Mb
Maker	*ab initio* plus evidence	23,145	23,145	6	854 bp	142 bp	138,596	19.8 Mb
Geneid	*ab initio*	62,259	62,259	2.9	553 bp	194 bp	177,361	34.5 Mb
Genscan	*ab initio*	32,320	32,320	3.5	844 bp	241 bp	112,777	27.3 Mb
Glean	consensus	36,606	36,606	4.3	943 bp	220 bp	156,578	34.5 Mb
GLEAN(-refseq)	consensus	24,355	24,355	2.8	657 bp	233 bp	68,632	16.0 Mb
OGS 1.0	NCBI RefSeq + non redundant GLEAN	34,604	34,821	4.3	1,024 bp	241 bp	148,081	35.7 Mb

NCBI RefSeq models are subdivided into 10,249 protein coding models completely or partially based on EST or protein alignments, plus 840 pseudogene models containing debilitating frameshift or nonsense codons and noncoding RNAs. For alternative transcripts, primary transcript variant in RefSeq and Augustus were used in mRNA/exon calculation. All exon calculations are based on coding sequences only. Average mRNA length does not include UTR sequences. OGS, Official Gene Set (RefSeq coding genes + non-redundant GLEAN).

*Ab initio* prediction requires the detection of intron/exon junctions based on rules observed from the major spliceosome machinery. However, some introns are excised by the minor spliceosome driven by the U12 small snoRNA, and these introns are poorly predicted by *ab initio* algorithms. We identified 134 putative U12 introns in the pea aphid genome representing the most identified in any insect. This high number of U12 introns likely complicates *ab initio* gene modeling in the pea aphid.

The combined total of 34,604 gene predictions includes unsupported *ab initio* models, partial gene models, and genes incorrectly shown as duplicated in the Acyr_1.0 assembly (see below). This estimate is likely, therefore, to exceed the true number of protein-coding genes. Nevertheless, the combined set of computational gene predictions provided a foundation for subsequent analyses, including manual annotation of 2,010 genes.

#### Genome-based phylogeny, genome comparisons, and gene phylogenies

**Figure 3:**
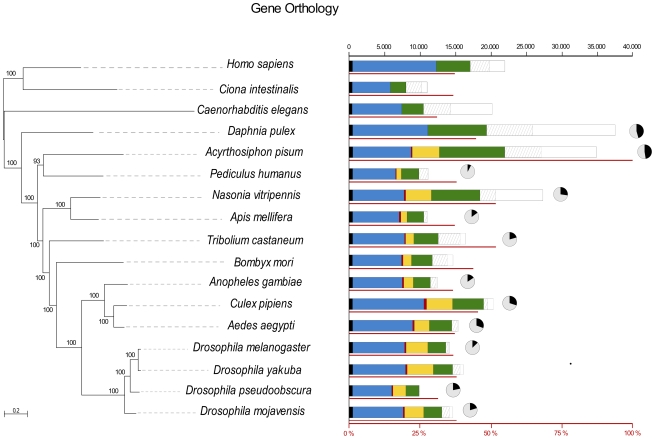
Comparative genomics across the insects. The phylogeny is based on maximum likelihood analyses of a concatenated alignment of 197 widespread, single-copy proteins. The tree was rooted using chordates as the most external out group. Bars represent a comparison of the gene content of all species included in the analysis (scale on the top). Bars are subdivided to indicate different types of homology relationships; black: widespread genes that are found with a one-to-one orthology in at least 16 of the 17 species; blue: widespread genes that can be found in at least 16 of the 17 species and are sometimes present in more than one copy; red: widespread but insect-specific genes present in at least 12 of the 13 insect species; yellow: non-widespread insect-specific genes (present in less than 12 insect species); green: genes present in insects and other groups but with a patchy distribution; white: species-specific genes with no (detectable) homologs in other species (striped fraction corresponds to species-specific genes present in more than one copy). The thin red line under each bar represents the percentage of *A. pisum* genes that have homologs in the given species (scale across the bottom of the figure). The fractions of single genes (grey) and duplicated genes (black) for some of the species are represented as pie charts.

We took advantage of the first genome for a hemipteran species to perform a whole genome-based species phylogeny of the insects. The resulting phylogeny, based on 197 genes with single copy orthologs, is congruent with previous phylogenetic analyses [42] and places the pea aphid together with *Pediculus humanus*, another member of the para-neoptera clade, basal to the Holometabola (Figure 3). Comparing gene content across this phylogeny revealed that the pea aphid shares 30%–55% (e-value<10^−3^) of its genes in its complete gene set with other sequenced insects, with the highest overlap with *Nasonia vitripennis* and *Tribolium castaneum* (53% in both cases) (Figure 3). However, 37% of predicted pea aphid genes have no significant hits (e-value<10^−3^) with genes identified to date in any other species. This large number of orphan genes may reflect high rates of false positive gene predictions or distinctive properties of the aphid genome, or both.

Beyond these comparisons—which are based on BLAST searches of aphid genes against other insect gene sets—we employed a phylogeny-based homology prediction pipeline [19],[43] to generate the pea aphid phylome: a phylogenetic tree and orthology prediction for every predicted, non-orphan *A. pisum* protein. Although rampant duplications have produced large gene families (see below), phylogeny-based orthology predictions allowed us to directly transfer GO annotations to 4,058 pea aphid genes that display one-to-one orthology relationships with annotated *Drosophila melanogaster* genes.

#### A wave of gene duplication

**Figure 4:**
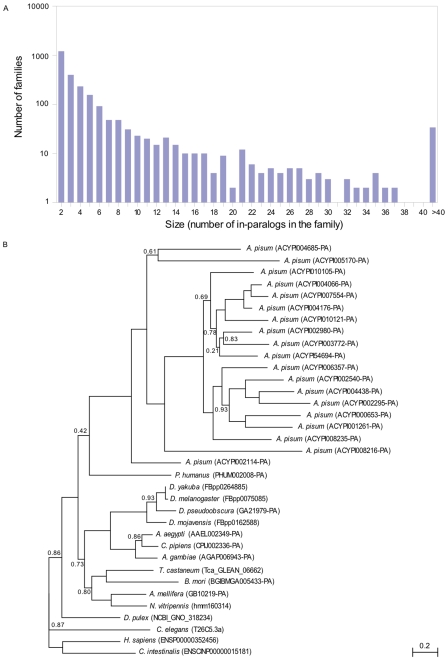
Lineage-specific gene expansions in the pea aphid. (A) Size distribution of the major lineage-specific groups of in-paralogs (i.e., paralogs resulting from duplications occurring after the split of the lineages leading to the pea aphid and the louse *Pediculus humanus*). The *y*-axis (logarithmic scale) represents the number of gene families with lineage-specific expansions of a given size (*x*-axis), as inferred from the pea aphid phylome. (B) Maximum likelihood phylogenetic tree showing lineage-specific expansion of a family coding for Acetyl-CoA transporter. This expansion has resulted in 19 paralogs in the pea aphid, whereas other insects and out groups included in the analysis possess only a single ortholog.

Analysis of the pea aphid phylome revealed 2,459 gene families that appear to have undergone aphid lineage-specific duplications, a number greater than that of any other sequenced insect genome (Figure 4A). Only the genome of the crustacean *Daphnia pulex* appears to have experienced a similar level of lineage-specific duplications [17]. The largest gene family expansions, involving 19 families with 50 to 200 members, encode reverse transcriptase and transposase domains probably representing pieces of transposable elements (TEs). However, most gene family expansions do not involve TEs. Notable examples include approximately 200 lineage-specific paralogs of the *Drosophila* gene *kelch*, which encodes an actin-binding protein involved in ovarian follicle cell migration and oogenesis (Gene tree ACYPI51424-PA in phylomeDB), and 19 paralogs of a putative Acetyl-CoA transporter (Figure 4B). This high level of gene duplication in the pea aphid genome is widespread among different types of genes, and numerous additional examples are discussed below.

**Figure 5:**
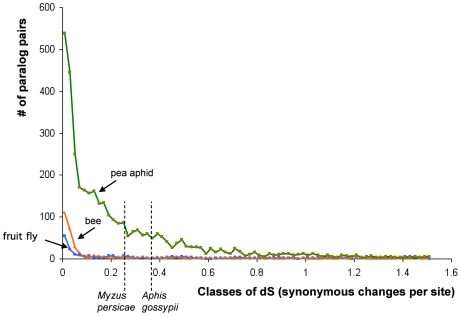
Widespread gene duplication in an ancestor of the pea aphid, as suggested by the frequency distribution of synonymous divergence (dS) between pairs of recent paralogs (Reciprocal Best Hits) within pea aphid, honey bee, and *Drosophila*. Vertical dotted lines show the estimated average dS between orthologs from different aphid species. 1: *A. pisum* and *Myzus persicae* (two species of the tribe Macrosiphini), mean dS = 0.25; 2: *A. pisum* and *Aphis gossypii* (tribe Aphidini), mean dS = 0.35 (estimates from [128]). Paralogs resulting from ancient duplications (dS>1.5) are also abundant in all three genomes (1,449 pairs in aphid, 1,726 in drosophila, 1,010 in bee; not shown).

To provide a time scale for the origin of aphid-specific duplications, we estimated the synonymous distances (dS values) among all paralog pairs, which were identified using a within-genome reciprocal best blast hit. Because the sequenced line showed some heterozygosity, divergence between truly paralogous gene pairs could be confounded with allelic variation, but this should be a problem only for very close pairs of paralogs, since divergence values for allelic variants in most systems are generally very low (<1%). The large majority of gene pairs have higher divergence (dS > 0.05) than this allelic variant cut-off value, and thus can be assumed to represent true paralogs. Paralog pairs display a wide range of dS values, suggesting that gene duplication has occurred for an extended time in the pea aphid lineage. The elevated gene duplication rate appears to have started early in aphid evolution, since the oldest paralog pairs within the pea aphid genome show dS values that are comparable to the dS values for ortholog pairs between pea aphid and *Aphis gossypii*, a species from a different aphid subfamily (Figure 5).

#### Telomeres

The pea aphid, similar to other non-dipteran insects, possesses a single candidate telomerase gene and the canonical arthropod telomere repeat of TTAGG [44]. Examination of raw read mate pairs revealed long stretches of TTAGG repeats at presumptive chromosome ends. Of the expected eight telomeres, we identified simple TTAGG repeats at the ends of five scaffolds: two contain relatively long repeat stretches of apparently true TTAGG simple repeat telomeres, while three are similar to the telomeres of *Bombyx* and *Tribolium* and contain non-LTR retrotransposon insertions [42],[45].

#### TEs

**Figure 6:**
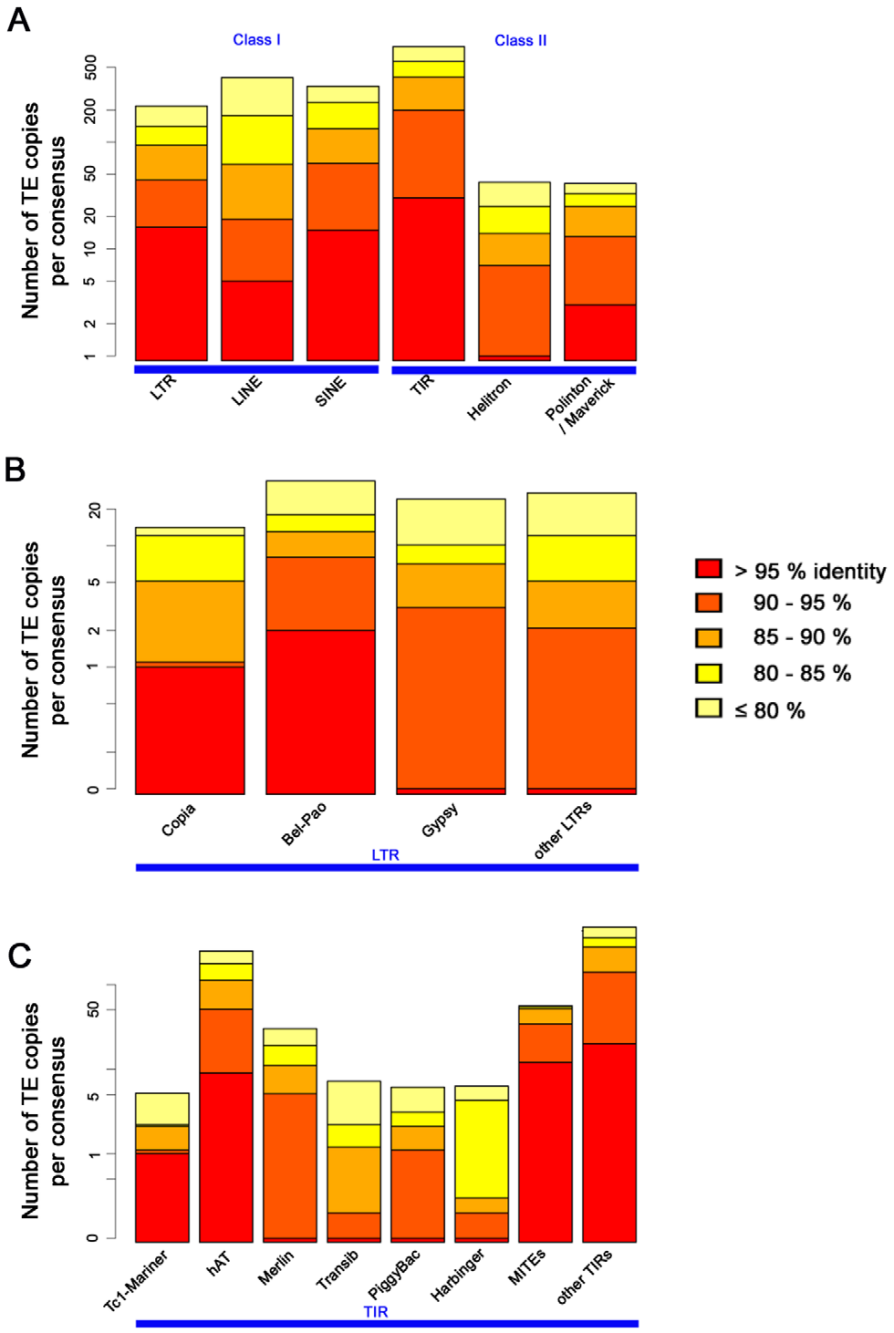
Transposable element copy identity distribution. We show the mean identities of (A) TE copies in the pea aphid genome to their consensus reference sequence, (B) LTR super-families, and (C) TIR super-families. The consensus reference TE sequences contain the most frequent nucleotide at each base position and are thus approximations of the ancestral TE sequences, correcting for mutations affecting a small number of copies. Hence, the identity here is a proxy for TE family ages, with recent family having high identity (few differences with the ancestral state), and allows the ordering of transposable element invasions of the pea aphid genome. Note that the repeat order “Others” (Table 1) is not shown here, and the *y*-axis is a log scale that emphasizes recent families.

Approximately 38% of the assembled genome is composed of TEs. We identified 13,911 consensus TE sequences in the pea aphid genome using REPET, a TE annotation pipeline. The consensus TE sequences were grouped by sequence similarity and classified according to their structural and coding features into 1,883 TE families (consisting of two or more consensus sequences) and 1,672 singletons. Within the 1,883 TE families, we manually curated 85 families including the largest families representative of widespread TE groups, such as LTRs, LINEs, SINEs, TIRs, and Helitrons (Table 2). The curated repeats account for 4% of the genome, and less complex repeat families with few sequence variants remain uncurated and account for 34% of the pea aphid genome. Of the curated repeats, most super-families represent old invasions, as indicated by the distribution of nucleotide identities between sequences within TE families (Figure 6).

**Table 2:** Repeat statistics of the curated and non-curated orders of transposable elements.

Order	Number of Families	Number of Curated Families	Number of Copies	Numbers of TE Copies for Curated Families	Coverage (% of the Genome)	Coverage of Curated Families (% Genome)
TIRs	320	38	46,155	11,063	4.382	1.656
LINEs	178	15	24,579	6,230	3.066	0.939
LTRs	69	17	11,199	5,405	1.365	0.741
SINEs	63	7	12,462	4,767	1.002	0.480
MITEs	20	3	5,104	2,461	0.420	0.250
Polintons	17	3	1,583	768	0.255	0.089
Helitrons	12	2	2,881	2,055	0.248	0.167
Others	1,216	NA	402,346	NA	27.117	NA
Total	1,883	85	506,309	32,749	37.856	4.321

Terminal inverted repeats (TIRs) and long interspersed elements (LINEs) are the most represented orders in the pea aphid genome. The repeat order named “Others” includes repetitive regions that match to pea aphid consensus TEs but could not be classified by the REPET pipeline because they lack structural features and similarities to other known TEs, and thus are not manually curated.

#### Chromatin modifications

Like the hymenopteran honey bee and parasitic wasp *Nasonia* and unlike other insects with sequenced genomes, the pea aphid has a full complement of DNA methylation genes, with orthologs for two maintenance DNA methyltransferases (*Dnmt1a* and *Dnmt1b*), two de novo DNA methyltransferases (*Dnmt3a* and *Dnmt3X*), and the *Dnmt2* found in all sequenced insect genomes. In addition to the DNA methyltransferases, we also identified a single putative methyl-DNA-binding-domain-containing gene involved in the recruitment of chromatin modification enzymes.

**Figure 7:**
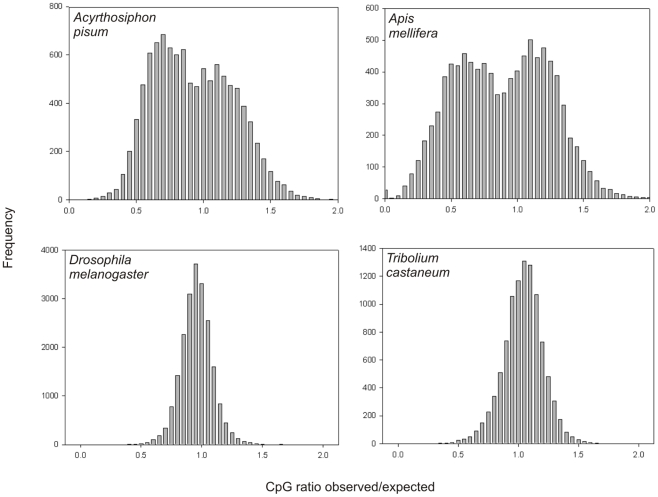
CpG ratios in the coding sequence of selected insects. CpG ratios were calculated using RefSeq data for each insect species. For each sequence the observed (obs) CpG frequency and the expected (exp) CpG frequency were calculated. The expected CpG frequency was calculated based on the GC content of each sequence and the CpG ratio was calculated as obs/exp. The frequency of each CpG ratio was plotted against the observed/expected ratio. A bimodal distribution was observed for *A. pisum* and *A. mellifera*, both of which show DNA methylation within the coding sequence of genes [37],[129]. *D. melanogaster* and *T. castaneum* both show a unimodal distribution, and there is only limited evidence of methylation in both of these species. In addition *A. pisum* and *A. mellifera* have all the DNA methyltransferases while *D. melanogaster* only has *Dnmt2* and *T. castaneum* has *Dnmt1* and *Dnmt2*.

Methylated C nucleotides in CpGs—the sites of known DNA methylation in pea aphid—are prone to deamination to uracil, after which DNA repair machinery can produce thymidine. Thus, an excess of CpG sites over those expected at random can provide evidence for purifying selection maintaining CpG sites for methylation. This approach has been used previously to successfully predict methylated genes [46]. We investigated the frequency in aphid genes of CpG sites compared with the frequency expected based on the low overall GC content. Pea aphids, like *Apis mellifera*, exhibit a double peak in the frequency of genes with different ratios of observed/expected CpG content, a pattern different than that of *Drosophila melanogaster* and of *Tribolium castaneum* (Figure 7). The double peak suggests two broad classes of genes with different methylation status. Direct examination of DNA methylation states will be required to confirm that two major groups of pea aphid genes are differentially regulated by methylation.

#### Small non-coding regulatory RNAs

**Figure 8:**
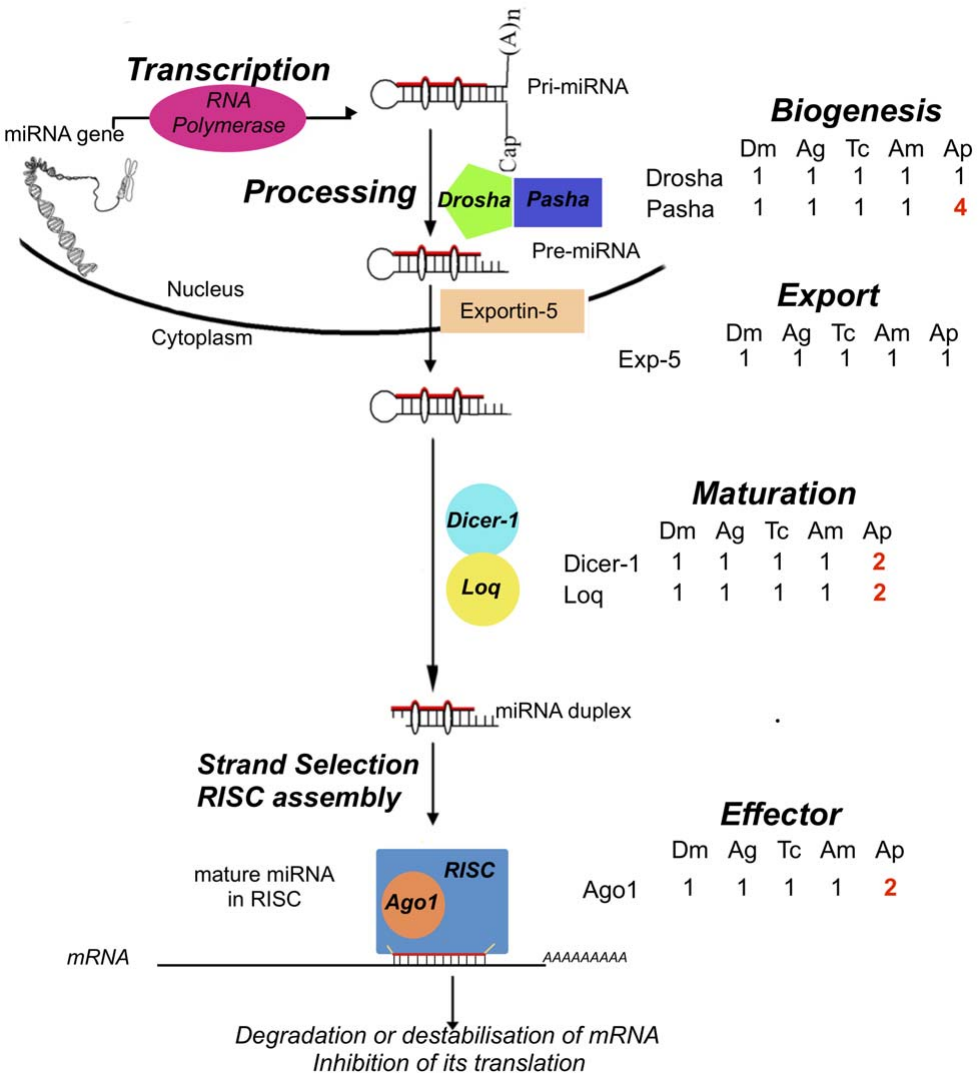
Expansion of the miRNA pathway in the pea aphid. miRNA biogenesis is initiated in the nucleus by the Drosha-Pasha complex, resulting in precursors of around 60–70 nucleotides named pre-miRNAs. Pre-miRNAs are exported from the nucleus to the cytoplasm by Exportin-5. In the cytoplasm, Dicer-1 and its cofactor Loquacious (Loq) cleave these pre-miRNAs to produce mature miRNA duplexes. A duplex is then separated and one strand is selected as the mature miRNA whereas the other strand is degraded. This mature miRNA is integrated into the multiprotein RISC complex, which includes the key protein Argonaute 1 (Ago1). Integration of miRNAs into RISC will lead to the inhibition of targeted genes either by the degradation of the target mRNA or by the inhibition of its translation. All components of the miRNA pathway have been identified in the pea aphid. Shown are the number of homologs in *A. pisum* (*Ap*) as well as *Drosophila melanogaster* (*Dm*), *Anopheles gambiae* (*Ag*), *Tribolium castaneum* (*Tc*), and *Apis mellifera* (*Am*). While all these genes are monogenic in these insect species, the pea aphid possesses two copies of *dicer-1*, *loquacious*, and *argonaute-1* and four copies of *pasha* (red font). The second *loquacious* copy is degraded and probably corresponds to a pseudogene.

Micro RNA and small interfering RNA gene silencing participates in regulation of eukaryotic gene expression [47]. We identified 163 microRNAs, including 52 conserved and 111 orphan microRNAs. We also found an expansion of gene families related to miRNA-related gene regulation (Figure 8). This expansion includes four copies of *pasha*, a co-factor of *drosha* involved in the first step of miRNA biosynthesis, a duplication of *dicer-1*, an RNAse involved in the processing of miRNAs, and a duplication of *Argonaute-1*, the key protein of the multiprotein RNA Induced Silencing Complex (RISC). These gene family expansions are present in other aphid species [21], but no other metazoa outside the aphids appear to have duplications of these genes.

### The Pea Aphid as a Host of Symbiont Bacteria

#### Genome of the primary symbiont *Buchnera aphidicola*

Most aphid species harbor the obligate, mutualistic, primary symbiont, *Buchnera aphidicola* (Gamma proteobacteria), within the cytoplasm of specialized cells called bacteriocytes. These bacteria are passed from mother to eggs during oogenesis in sexual forms and directly to developing embryos during embryogenesis of asexual morphs [48].

Although this sequencing project was designed to target the genome of *A. pisum*, the project also generated sequences of the primary symbiotic bacteria, *Buchnera aphidicola* APS. We obtained 24,947 sequence reads corresponding to ∼20× coverage of the *Buchnera* genome. Assembly of this sequence and PCR-based gap closure allowed reconstruction of the complete 642,011-base-pair genome of *Buchnera* (Genbank Accession ACFK00000000). Compared with the first sequenced strain from Japan [10], the new strain (from North America) shows approximately 1,500 mismatches (0.23%) and two larger inserts (1.2 kbp and 150 bp). The newly sequenced strain is almost 100% identical to a cluster of five recently sequenced *Buchnera* strains from pea aphids collected in North America (CP001161; [49]).

Besides *Buchnera*, aphids often harbor facultative heritable symbiotic bacteria known as secondary symbionts, of which different strains have been shown to protect pea aphid hosts from heat stress, fungal pathogens, and parasitoid wasps [6]. As part of the pea aphid genome project, the genomic sequence of the secondary symbiont *Regiella insecticola* was obtained [8]. Along with the recently completed sequence for the secondary symbiont *Hamiltonella defensa* [9], these data contrast with the genomes of *Buchnera* and other obligate symbionts, illustrating the genomic underpinnings of two very different symbiotic lifestyles. *Buchnera* possesses a highly reduced genome largely comprised of genes essential for basic cellular processes and aphid nutrition. Its chromosome is unusually stable and completely lacks mobile elements, bacteriophage, or genes for toxin production. In contrast, *H. defensa* and *R. insecticola* possess phage genes, many mobile elements, and numerous genes predicted to encode toxins [6],[8],[50]. For example, about 12% of all *R. insecticola* genes are homologous to transposases of mobile elements, and 5% of genes are phage-related, suggesting a highly dynamic genome especially as compared to *Buchnera* and other small genome symbionts.

#### Lateral gene transfer from bacteria to the host

The pea aphid genome provides a first opportunity for an exhaustive search for genes of bacterial origin in the genome of a eukaryotic host showing persistent associations with heritable bacterial symbionts. Besides their ancient association with *Buchnera* and facultative associations with *Regiella* and other symbionts within the Enterobacteriaceae [51], aphids sometimes harbor *Spiroplasma* species, *Rickettsia* species, and *Wolbachia* species as heritable endosymbionts.

Screening of the genome project data for bacterial sequences revealed a large number of genes of apparent bacterial origin, even after vector contaminants had been screened out. However, a majority of these were on small contigs (mostly under 5 kb) that did not contain evident aphid sequence; PCR experiments on a subsample of such genes supported their identity as bacterial contaminants in the dataset rather than as true transferred genes (Table S5). A minority of apparent bacterial genes was present on larger contigs, some of which contained genes of evident insect origin, suggesting that these represented true transferred genes. Phylogenetic analyses, incorporating homologous genes from prokaryotes and eukaryotes, supported the bacterial origin of 12 such genes or gene fragments, extending previous findings of gene transfer from a bacterial lineage to the aphid genome [52],[53]. Apparent transferred genes included those encoding LD-carboxypeptidases (LdcA), *N*-acetylmuramoyl-L-alanine amidase (AmiD), 1,4-beta-*N*-acetylmuramidase, and rare lipoprotein A (RlpA). Several of the genes originating from bacteria were previously detected as transcripts expressed in bacteriocytes [52], where some are highly expressed [53]. The coding regions of most of these genes are intact. Another source of transferred DNA is the mitochondrial genome, and aphids were one of the first animals for which transferred mitochondrial genes were reported [54]. In the pea aphid genome, a total of 56 mitochondrial gene sequences were detected. All of these transferred mitochondrial genes have been pseudogenized through substitutions and deletions, and some transferred sequences have been duplicated.

Our findings indicate that overall aphids have acquired few functional genes via lateral gene transfer from bacteria. However, these few genes may be critical in the maintenance of the symbioses exhibited by aphids.

#### Metabolism and symbiosis

The pea aphid genome provides insight into the intimate metabolic associations between an insect host and obligate bacterial symbiont, revealing how the pea aphid's amino acid and purine metabolism might be adapted to support essential amino acid synthesis and nitrogen recycling by *Buchnera*. Manual annotation of metabolism genes reveals that, like other animals, the pea aphid lacks the capacity for de novo synthesis of nine protein-amino acids (histidine, isoleucine, leucine, lysine, methionine, phenylalanine, threonine, tryptophan, and valine). All genes underlying the urea cycle are also missing, rendering the pea aphid incapable of synthesizing a further amino acid, arginine.

**Figure 9:**
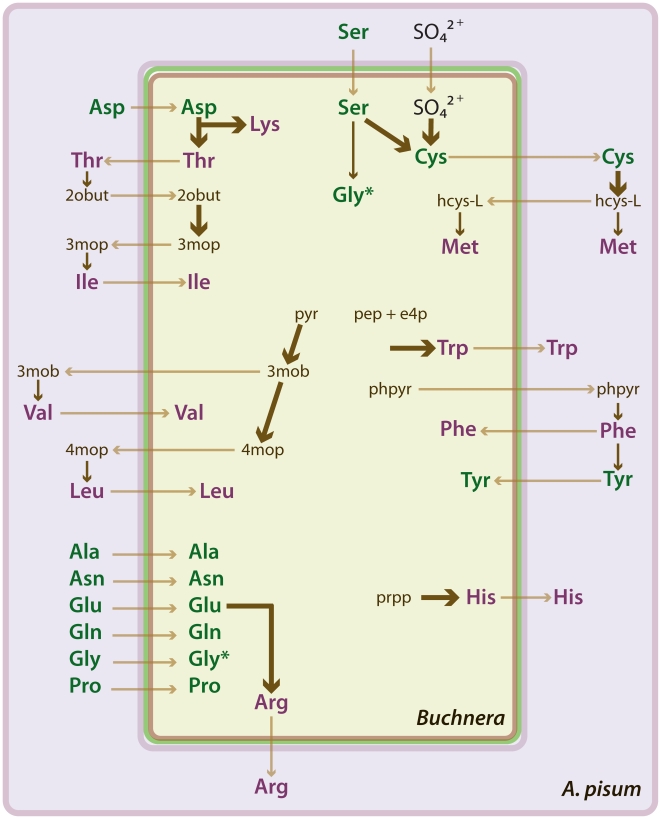
Amino acid relations of the pea aphid *Acyrthosiphon pisum* and its symbiotic bacterium *Buchnera aphidicola*. The schematic shows hypothetical relations based on the annotation of amino acid biosynthesis genes in the two organisms. *Buchnera* cells are located in the cytoplasm of specialized aphid cells, known as bacteriocytes. Each *Buchnera* cell is bound by three membranes, interpreted as the inner bacterial membrane (brown), outer bacterial membrane (green), and a membrane of insect origin known as the symbiosomal membrane (purple). The predicted biosynthesis (dark arrows) of essential amino acids (purple) and nonessential amino acids (green) and transport (light arrows) of metabolites between the partners are shown. The thickness of dark arrows indicates the number of metabolic reactions represented; thin arrows represent a single reaction and thick arrows more than one reaction. *The amino acid Gly appears twice in the *Buchnera* cell because it is synthesized by both *Buchnera* and the aphid (and possibly taken up by *Buchnera*). Metabolite abbreviations appear as follows: 2obut, 2-oxobutanoate; 3mob, 3-methyl-2-oxobutanoate; 3mop, (S)-3-methyl-2-oxopentanoate; 4mop, 4-methyl-2-oxopentanoate; e4p, D-erythrose 4-phosphate; hcys-L, homocysteine; pep, phosphoenolpyruvate; phpyr, phenylpyruvate; prpp, phosphoribosyl pyrophosphate; pyr, pyruvate.

A global view of the metabolism of the pea aphid as inferred from genome sequence data is available at AcypiCyc, a dedicated BioCyc database (see http://pbil.univ-lyon1.fr/software/cycads/acypicyc/home and Table S6) [55]. This analysis highlighted several noteworthy features of pea aphid metabolism. First, the genetic capacities of pea aphids and of *Buchnera* for amino acid biosynthesis are broadly complementary, an effect that can be attributed principally to gene loss from *Buchnera* [10],[56]. This complementarity results in several apparent instances of metabolic pathways shared between the pea aphid and *Buchnera* (Figure 9). For example, the aphid genome includes a gene for glutamine synthetase 2, which is highly expressed in the bacteriocytes that house *Buchnera* [52]. This raises the possibility that bacteriocytes actively synthesize glutamine, which is then utilized by *Buchnera* as an amino donor in several metabolic pathways, including arginine synthesis. Second, the pea aphid apparently lacks two core genes of the purine salvage pathway, adenosine deaminase and purine nucleoside phosphorylase, as well as genes necessary for the urea cycle. The absence of these genes makes it unlikely that aphids can produce uric acid or urea, an inference consistent with the absence of detectable uric acid or urea in pea aphid excreta [57].

Analyses revealed an additional unusual trait with implications for metabolism. Neither the aphid nor *Buchnera* has the genetic capacity to utilize selenocysteine, the 21st protein amino acid. Selenocysteine is encoded by the codon UGA, normally a stop codon. A number of specific genes and factors comprise the selenoprotein machinery required to recode UGA to selenocysteine [58]. Although cysteine homologs were found for some selenoproteins, no homolog was found for the known insect selenoproteins, nor did we find a tRNA for selenocysteine. Additionally we searched for the selenoprotein machinery genes (*SBP2*, *Efsec*, *Secp43*, *pstk*, *SecS*, *SPS1*, and *SPS2*) and found only *SPS1,* which appears to not function in selenocysteine biosynthesis in insects [59] and *SecS*. *Buchnera* does not have the genetic capacity to compensate for these gene losses. Together, these findings strongly suggest that *A. pisum* lacks the capacity to make selenoproteins, a trait atypical for an animal [60],[61].

#### Immune system of an animal with an obligate bacterial symbiosis

**Figure 10:**
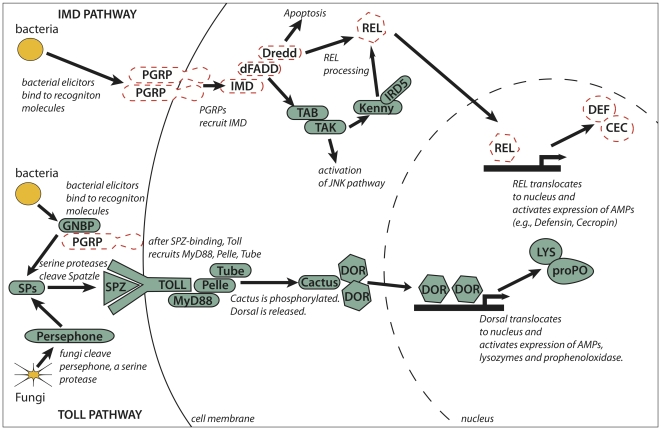
The IMD immune pathway is missing in the pea aphid. Previously sequenced insect genomes (fly, mosquitoes, honeybee, red flour beetle) have indicated that the immune signaling pathways, including IMD and Toll pathways shown here, are conserved across insects. In *Drosophila*, response to many Gram-negative bacteria and some Gram-positive bacteria and fungi relies on the IMD pathway. In aphids, missing IMD pathway genes (dashed lines) include those involved in recognition (PGRPs) and signaling (IMD, dFADD, Dredd, REL). Genes encoding antimicrobial peptides common in other insects, including defensins and cecropins, are also missing. In contrast, we found putative homologs for all genes central to the Toll signaling pathway, which is key to response to bacteria, fungi, and other microbes in *Drosophila.*

The aphid immune system is expected to be critical in determining responses to microbial symbionts [62]. Orthologs of the key components of the immune-related Toll, Jak/Stat, and JNK signaling pathways are present in the pea aphid genome. However, other immune response pathways appear to be absent (Figure 10). Specifically, many of the genes comprising the IMD (Immunodeficiency) pathway, including *IMD*, *dFADD*, *Dredd*, and *Relish*, could not be detected in the pea aphid genome. The IMD pathway is intact in genomes of other sequenced insects [63], and some of these IMD pathway genes are found in the crustacean, *Daphnia pulex* [64]. Furthermore, the pea aphid genome also lacks recognizable peptidoglycan recognition proteins (PGRPs), which detect certain pathogens and trigger the IMD and Toll pathways in *Drosophila* [62]. Additionally, manual annotation identified few antimicrobial peptide (AMP) genes, which are produced in response to activated immune pathways. Consistent with this, studies of immune-challenged pea aphids—using a variety of assays (SSH, ESTs, HPLC) that have successfully identified AMP genes in other species—recovered no AMPs from bacteria-challenged or fungal-challenged aphids [16],[65]. These studies found that during immune challenges, aphids up-regulate few genes of known immune function and few novel genes that could be associated with an alternate immune response. Together our observations suggest that, in comparison to previously studied insects, aphids have a reduced immune repertoire. Reduced immune capabilities could facilitate the acquisition and maintenance of microbial symbionts, a hypothesis testable in other obligately symbiotic systems. An alternate possibility is that rapid reproduction and a largely microbe-free diet of phloem sap, decrease selective pressures on the aphid to maintain costly immune protection.

### Genome of a Phloem-Feeding Specialist

#### Finding a suitable host plant

Plant volatiles are important cues for host plant recognition by aphids. In insects, such cues enter the antennae, bind to odorant-binding proteins (OBPs) [66],[67] and are transported to chemoreceptors [68],[69],[70],[71], which then activate a cascade of events leading to sensory neuron activity. Chemoreceptors include basal gustatory receptors (GRs) and more derived odorant receptors (ORs). Chemosensory proteins (CSPs) are also thought to be involved in chemoreception.

We identified 15 genes encoding putative OBPs and 13 putative CSP genes. By way of contrast, other insects also have more OBPs than CSPs [72]. Zhou et al. (2009) also identified highly conserved orthologs for 10 of the 15 pea aphid OBPs in nine other aphid species [39].

We identified 79 genes in the OR family, including intact, partially annotated genes, and putative pseudogenes. An ortholog of the highly conserved *DmOr83b* gene [73] was named *ApOr1*. As in other sequenced genomes, the remainder of the OR genes represent aphid-specific expansions with no orthologs in other insects.

The pea aphid GR family contains at least 77 genes. There are six members of the well-conserved sugar receptor subfamily and no homologs of the highly conserved carbon dioxide receptors found in holometabolous insects [74]. The remaining 71 GR genes are orphans. Overall, the number of the OR and GR chemoreceptor classes does not differ substantially from that seen in other insects. Smadja et al. found that for both the OR and GR genes, some subfamilies appear to have resulted from relatively old duplication events, whereas others represent recent duplication events [34]. The rapid evolution of some OR and GR genes might be related to host plant specialization observed in *A. pisum* (for example, [75],[76]), because host plant acceptance has been shown to rely mainly on chemosensory processes [77].

#### Virus transmission

Responsible for transmission of 28% of known plant viruses, aphids show four modes of virus transmission; (1) non-persistent (stylet-borne), (2) semi-persistent (foregut-borne), (3) persistent circulative, and (4) persistent propagative [78]. The persistent circulative mode of transmission is exploited by members of the *Luteoviridae* family, which are transmitted specifically by aphids. Because luteovirids are transported by membrane trafficking mechanisms, proteins involved in endocytosis, vesicle transport, and exocytosis are potentially involved in virus transmission. As expected, we found genes for such proteins in the pea aphid genome. Of particular interest, we found 12 genes encoding a novel type of dynamin, which are large GTPases involved in membrane dynamic processes.

#### Detoxification of plant defenses

As an herbivore, the pea aphid is likely to overcome plant chemical defenses, at least in part, by employing detoxification enzymes, including cytochrome P450 monooxygenases (P450s), glutathione *S-*transferases (GSTs), and carboxyl/choline esterases (CCEs). From the genome sequence, 83 potential pea aphid P450 genes have been identified, but only 58 of these have a complete P450 domain and good homology to other insect P450s. Although previously studied insects harbor six classes of GSTs [79], the 20 identified pea aphid GSTs belong to only three of these classes. The CCE gene family has 29 members in the pea aphid, all of which appear to encode functional proteins. Although the pea aphid has fewer detoxification enzymes than the non-herbivorous insects whose genomes have been examined (*Drosophila*, *Anopheles*, and *Tribolium*), it possesses more than the pollinator *Apis mellifera* [40].

#### Using phloem sap, a sugar-rich food source

The osmotic pressure of phloem sap is significantly greater than that of aphid hemolymph [80], and thus sugar transport can occur down a concentration gradient. Consistent with this we find that sodium-sugar symporters, proteins that facilitate movement against concentration gradients, are absent from the pea aphid genome. Instead, sugar transport from gut to hemolymph apparently relies on uniporters, proteins that exploit favorable concentration gradients to transport sugars from the gut into epithelial cells, and from epithelial cells into the hemolymph. The pea aphid genome contains a large number of uniporter-encoding genes, including approximately 200 genes encoding proteins of the major facilitator superfamily (MFS). Companion work [28] found that the most abundant sugar transporter transcript encodes a uniporter with capacity to transport both fructose and glucose. The pea aphid with 34 sugar/inositol transporter genes has more than *Drosophila melanogaster* (15 genes), *Apis mellifera* (17 genes), *Anopheles gambiae* (22 genes), and *Bombyx mori* (19 genes), but less than *Tribolium castaneum* (54 genes) [28]. Among these 34 pea aphid sugar/inositol transporter genes, 8 occur as either tandem repeats or inverted repeats, suggesting that they may have resulted from recent duplication events. Adaptation of aphids to an “extreme” diet requiring specialized sugar transport has likely contributed to the evolutionary expansion of this gene family.

### Development in a Polymorphic Insect

#### Overview of development

As hemimetabolous insects, aphids undergo incomplete metamorphosis, passing through a series of molts involving four immature instars to reach the adult stage. Aphids display a wide range of adult phenotypes (Figure 1) and possess two divergent modes of embryonic development: parthenogenetic and sexual embryogenesis [48].

#### Embryogenesis

The majority of genes involved in axis formation, segmentation, neurogenesis, eye development, and germ-line specification in the embryo are well-conserved. Genes playing critical roles in *Drosophila* embryogenesis, but thus far not found outside the Diptera, are also missing from aphids, including *oskar* (germ-line specification), *bicoid* (anterior development), and *gurken* (dorso-ventral patterning). Despite the absence of these orthologs, the downstream components of the developmental pathways to which they belong are well-conserved. Lineage-specific gene losses were found for *giant*, *huckebein*, and *orthodenticle-1.* Orthologs of some genes involved in establishing the body plan, such as *spätzle* and *dorsal*, have undergone aphid-specific gene duplications. There are also two paralogs of *torso-like*, the gene encoding the most conserved molecule in the terminal patterning pathway.

#### Chitin-related proteins

In arthropods, chitin contributes to the structure of the cuticle (i.e., the lining of the tracheae, foregut, and hindgut; and the exoskeleton). There are three major classes of chitin-binding proteins. The pea aphid genome contains a large expansion of the first class, genes containing the R&R consensus sequence [81], and multiple copies of the second class, genes with a cysteine-based chitin-binding domain (CBD). For the third class, genes containing a chitin deacetylase domain, the pea aphid genome encodes five of the six main types. Consistent with the aphid's lack of a peritrophic membrane, the sixth type, which is located in the peritrophic membrane of other insects, is absent in the pea aphid. Compared to other insects, the pea aphid has fewer genes encoding chitinase, an enzyme with chitinolytic activities that degrades old cuticle. This difference possibly reflects the fact that hemimetabolous insects, which do not undergo a complete metamorphosis to the adult form, do not require dramatic exoskeletal reconstruction.

#### Signaling pathways and transcription factors

Genes of the highly conserved TGF-β, Wnt, EGF, and JAK/STAT signaling pathways, all utilized in development, have undergone several aphid-specific duplications and losses. Multiple paralogs of *Dpp* (4 paralogs), *Medea* (5), *Mad* (2), *Domeless* (4), *STAT* (2), *Argos* (4), and *Armadillo* (2) are found in the pea aphid genome. These gene expansions are of particular note because duplications of genes that encode the components of signaling pathways are rare in animals [82]. Conversely, we identified aphid lineage-specific gene losses for several TGF-β ligands (*BMP10, Maverick*, and *Alp23*), Wnt ligands (Wnt6, Wnt10), and *Sprouty* (RTK signaling inhibitor).

The pea aphid genome contains 640 putative sequence-specific transcription factors. Most of the transcription factor families are similar in size and composition to those of other insects. However, the pea aphid genome encodes significantly more zinc-finger-containing proteins than other insects with sequenced genomes. Although the number of bHLH encoding genes is similar to other insects, orthologs of the *achaete-scute* genes, which are required for neurogenesis and bristle development in *Drosophila* and are found in other (holometabolous) insect genomes, were not found. All Hox complex genes are present, but *Hox3 (zen)* and *ftz*, which have evolved non-homeotic functions in insects, are highly divergent from the orthologs of other species.

#### Juvenile hormone (JH)

JH has been implicated in regulating aphid reproductive polyphenism [83],[84]. The main enzymes responsible for the synthesis and degradation of JH are present in the pea aphid genome, and several of these developmental genes are methylated [37], supporting the hypothesis that methylation could play a role in the developmental plasticity of aphids as it does in other insects (Table 3). The pea aphid apparently lacks other JH associated proteins such as hexamerins, which constitute a class of JH binding proteins implicated in many physiological processes including caste regulation of lower termites [85].

**Table 3:** Juvenile hormone related genes in the pea aphid genome exhibit different states of CpG methylation.

Gene Name	Abbreviation	Pea Aphid Gene Prediction	Pea Aphid CpG Methylation	*Drosophila Melanogaster*	*Tribolium Castaneum*	*Apis Mellifera*	*Bombyx Mori*
Juvenile Hormone Acid Methyltransferase	JHAMT	ACYPI255574	Not found	FBgn0028841	NM_001127311	XM_001119986	NM_001043436
		ACYPI568283	Not found				
Cytosolic Juvenile Hormone Binding Protein	JHBP	ACYPI154871	**Detected**		XM_964351	XM_625097	NM_001044203
Juvenile Hormone Epoxide Hydrolase	JHEH	ACYPI275360	Not found	FBgn0010053	XM_970006	XM_394354	NM_001043736
		ACYPI189600	Not found	FBgn0034405		XM_394922	
		ACYPI307696	**Detected**	FBgn0034406			
Juvenile Hormone Esterase^a^	JHE	ACYPI381461	Not examined				
Juvenile Hormone Esterase Binding Protein	JHEBP	ACYPI563350	**Detected**	FBgn0035088	XM_964394		NM_001047009
Hexamarin	Hex	No homolog			XM_961866	NM_001110764	
					XM_962135	NM_001098717	
						NM_001101023	
Methoprene-tolerant	Met	hmm126914	Not examined	FBgn0002723	NM_001099342		NM_001114986
Allatostatin	Ast	hmm252834	Not examined	FBgn0015591	XM_001809286		NM_001043571
Allatostatin receptor		ACYPI008623	Not examined	FBgn0028961		XM_397024	NM_001043570
FKBP39		ACYPI003035	Not examined				
Chd64		ACYPI003572	Not examined	FBgn0035499		XM_392114	
Broad	Br	ACYPI008576	Not examined	FBgn0000210	XM_001810758	NM_001040266	NM_001043511
					XM_001810798	XM_393428	
Retinoid X receptor (ultraspiracle)	RXR (usp)	ACYPI005934	Not examined	FBgn0003964	NM_001114294	NM_001011634	NM_001044005

a. The predicted juvenile hormone esterase is identified by the characteristic GQSAG motif and does not show significant homology to other known JHEs.

#### Mitosis, meiosis and cell cycle

**Figure 11:**
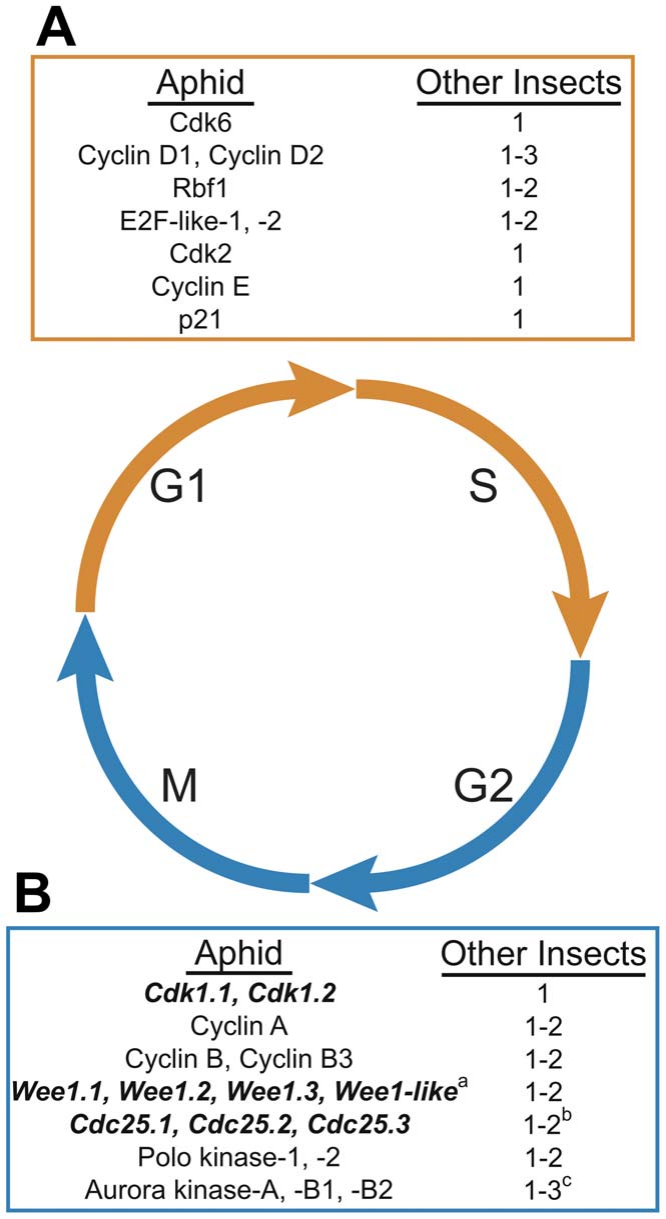
Kinases important in the regulation of mitosis have expanded in the pea aphid genome. The cell division cycle typically consists of four phases: two growth phases (G1 and G2), a DNA synthesis or replication phase (S), and mitosis (M). Distinct and overlapping sets of regulatory genes are required for orderly progression through these phases. (A) Genes important for G1 and S phase progression are similar in number to other insects (orange box). G1/S Cyclin/Cyclin-dependent kinase (Cdk) protein complexes, along with E2F transcription factors, are critical for entry into G1 and progression into DNA replication and are opposed by cell cycle inhibitors such as p21/p27 family members and pRb/p107 family (Rbf) members, respectively. (B) Genes important for G2 and M phases have expanded in pea aphids (blue box). Polo kinases, Aurora kinases, Cdc25 phosphatases, and G2/M Cyclin/Cdk protein complexes are all critical for promoting entry into and progression through mitosis and meiosis. Negative regulators of Cdk1 and entry into mitosis include the Wee1/Myt1 kinase family. However, while Cdk1 has undergone aphid-specific duplication, no expansion of its activation subunits, Cyclins A and B, has been observed. Expanded gene families are in bold italics. Copy number was compared to that in *Drosophila melanogaster*, *Tribolium castaneum*, *Pediculus humanas*, *Nasonia vitripennis*, *Culex quinquefasciatus*, *Anopheles gambiae*, *Aedes aegyptii*, *Bombyx mori*, and *Apis mellifera*. ^a^No Myt1 orthologs were identified in the *A. pisum* genome. ^b^Among sequenced insects other than the pea aphid, Cdc25 is duplicated only in Drosophilids. ^c^Three Aurora kinase orthologs are also present in *Nasonia* and *Aedes* while other insects possess two orthologs.

Aphids exhibit plasticity in meiosis and the cell cycle, allowing for both sexual reproduction and parthenogenesis. Most genes involved in meiosis and the cell cycle in vertebrates and yeasts are present in the pea aphid genome, while other sequenced insect genomes show lineage-specific losses of individual genes or gene family members [86]. While genes known to regulate the transition from G1 (growth) to S (DNA replication) phases of the cell cycle in metazoans are present in aphids (Figure 11A), the pea aphid genome also contains lineage-specific duplications of several mitotic regulators, such as *Cdk1*, *Polo*, *Wee1*, *Cdc25*, and *Aurora* (Figure 11B). In addition, the pea aphid genome contains lineage-specific duplications of several mitosis-related genes, including *Smc6* (structural maintenance of chromosomes 6) and *Topo2* (DNA Topoisomerase 2). These genes are single copy in other insects with sequenced genomes but duplicated in the Crustacean, *Daphnia pulex*, which is also capable of both sexual and asexual reproduction [87].

#### Neuropeptides, biogenic amines, and their receptors

Neuropeptides and biogenic amines are cell-to-cell signaling molecules that act as hormones, neurotransmitters, and/or neuromodulators [88]. By homology search, we found 42 genes encoding at least 70 neuropeptides and neurohormones. Expressed sequence tag and proteomic analyses suggest that many of these genes are active [20]. The *vasopressin* (which in insects is called inotocin, from insect oxytocin/vasopressin-related peptide; [89]), *sulfakinin*, and *corazonin* precursor genes and their respective receptors were not found. *Corazonin* has been found previously in several hemipteran species [90] and is involved in the regulation of migratory phase transition in *Locusta* and *Schistocerca* [91]. The pea aphid is the first sequenced insect genome lacking a *sulfakinin* gene. We found 18 biogenic amine G protein-coupled receptor (GPCR) genes and 42 genes encoding neuropeptide and protein hormone GPCRs. In general, there is excellent agreement between the presence or absence of neuropeptides and the presence or absence of their GPCRs.

#### Circadian rhythm

**Figure 12:**
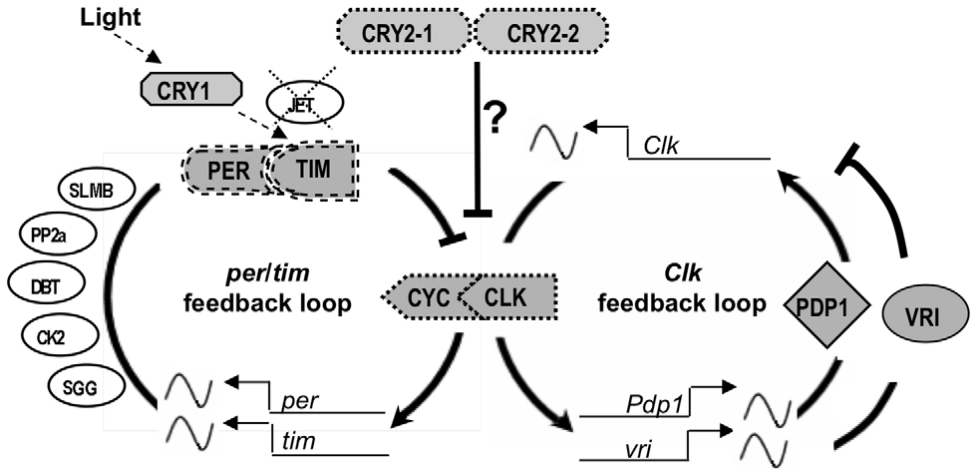
Orthologs of circadian clock genes, some significantly diverged, are found in the pea aphid genome. Shown is a schematic representation of pea aphid orthologs of the circadian clock genes arranged in a two-loop model, as proposed for *Drosophila* [92],[130]. Genes constituting the core of the clockwork in *Drosophila* are in filled shapes; other genes relevant to the clock mechanism in *Drosophila* are in empty ovals. In *Drosophila*, the *per*/*tim* feedback loop is centered on the transcription factors PER and TIM encoded by the genes *period* (*per*) and *timeless* (*tim*). *Kinase 2* (CK2) and *Shaggy* (SGG), the *Protein phosphotase 2a* (PP2A), and the degradation signaling proteins *Supernumerary limbs* (SLMB) and *jetlag* (JET) participate in this loop either by stabilizing or destabilizing PER and TIM. Light entrainment is mediated through the participation of *Cryptochrome 1* (CRY1) and JET, which promote the degradation of TIM. Absence of JET in *A. pisum* is indicated by a dashed cross. The positive feedback loop in *Drosophila* is centered on the gene *Clock* (Clk), whose expression is regulated by the products of the genes *vrille* (VR1) and *Pdp1* (PDP1). In addition to all these genes, the pea aphid genome contains two copies of a mammalian-type cryptochrome, CRY2, which is present in all other insects examined except *Drosophila*. CRY2 has been proposed to be part of the core mechanism [93], acting as a repressor of CLK/CYC (indicated by a question mark). Some pea aphid orthologs have diverged significantly compared with orthologs in other insects (dashed outlines). This is most dramatic for PER and TIM proteins (double dashed outlines), whose sequences differ significantly from those of other insects. Wavy lines indicate rhythmic transcription in *Drosophila*. Thick arrows and lines ending in bars indicate positive and negative regulation, respectively.

Circadian clocks are internal oscillators governing daily cycles of activity and are proposed to underlie responses to day-night cycle, the most important cue triggering aphid reproductive polyphenism. In *Drosophila*, the circadian clock is regulated by two interdependent transcriptional feedback loops involving several genes of which the genes *period* and *clock* occupy a central position [92]. All core genes from both loops were found in the pea aphid genome (Figure 12). The pea aphid *Clock* feedback loop shows high conservation of *Clock*, *Vrille*, and *Pdp1*. In contrast the *period/timeless* feedback loop is not well conserved. Two other participants at the core of the circadian clock, the cryptochromes *Cry1* and *Cry2* [93], are present in the pea aphid genome. *Cry2*, which is absent in *Drosophila* but present in single copy in all non-drosophilid insects, is duplicated in *A. pisum*, a pattern similar to that found in many vertebrates. Additional genes required for the *Drosophila* circadian clock, including the kinases *double-time*, *shaggy*, *casein kinase 2*, *protein phosphatase 2a*, and the protein degradation protein *Supernumerary Limbs*, are found in the pea aphid genome. We did not detect the F-box protein *jetlag*, which is necessary for light entrainment in *Drosophila* (Figure 12) [94].

#### Sex determination

Aphid sex determination is chromosomal. Females have two X chromosomes and males have only one [95]. We searched the *A. pisum* genome for homologs of 32 sex-determination-related genes previously characterized in *Drosophila melanogaster*. Of the 32 genes, pea aphid homologs of 22 (69%) were identified. Like the honeybee, the pea aphid has homologs of the penultimate gene (*transformer 2*) and the DM-DNA binding domain of the ultimate gene (*doublesex*) genes of the *D. melanogaster* sex determination pathway. Multiple hits to four of the 32 genes were found in the pea aphid, all representing recent duplication events.

### Concluding Remarks

Major results from analyses of the pea aphid genome can be summarized as follows:

- Extensive gene duplication has occurred in the pea aphid genome and appears to date to around the time of the origin of aphids.
- The aphid genome appears to have more coding genes than previously sequenced insects, although a precise gene count awaits better assembly and further functional annotation of the genome. The increased gene number reflects both extensive duplications and the presence of genes with no orthologs in other insects.
- More than 2,000 gene families are expanded in the aphid lineage, relative to other published genomes; examples include families involved in chromatin modification, miRNA synthesis, and sugar transport.
- Orphan genes comprise 20% of the total number of genes in the genome. Many are found in EST libraries, suggesting they are functional.
- As the first genome sequenced for an animal with an ancient coevolved symbiosis, the pea aphid genome reveals coordination of gene products and metabolism between host and symbionts. Amino acid and purine metabolism illustrate apparent cases of biosynthetic pathways for which different enzymatic steps are encoded in distinct genomes. These preliminary findings of host-symbiont coordination will be enhanced by the availability of genomes for three pea aphid symbionts, including the obligate nutritional symbiont *Buchnera*.
- Selenocysteine biosynthesis is not present in the pea aphid, and selenoproteins are absent.
- Several genes were found to have arisen from bacterial ancestors. Some of these genes are highly expressed in bacteriocytes and may function in regulation of the symbiosis with *Buchnera.*
- The immune system of pea aphids is reduced and specifically lacks the IMD pathway; this unusual loss may be linked as a cause or consequence of the evolution of intimate bacterial symbioses.
- As a specialized herbivore, the pea aphid must overcome plant defenses, and the pea aphid genome provides candidates for genes involved in critical insect-plant interactions.
- The unusual developmental patterns of aphids, involving extensive polyphenism, may be facilitated by duplications of many development-related genes.

Our analysis of the pea aphid genome has begun to reveal the genetic underpinnings of this animal's complex ecology—including its capacity to parasitize agricultural crops, its association with microbial symbionts, and its developmental patterning. One project benefiting from the availability of the genome sequence is the investigation of aphid saliva proteins [12] thought critical for host plant feeding. This highlights the ability of the genome to facilitate future exploration of both basic and applied biological problems.

## Materials and Methods

### Sequencing Strain

The parental line of the sequenced aphid clone, LSR1, was collected in a field of alfalfa (*Medicago sativa*) near Ithaca, New York, in 1998 [96]. Aphids for DNA isolation resulted from a single generation of inbreeding to produce LSR1.AC.G1. The LSR1.AC.G1 aphid line was grown from a single female and treated with ampicillin to remove *R. insecticola*. Prior to DNA preparation, aphids were heat treated to reduce the number of *Buchnera* cells; entire aphid colonies on broad bean plants were placed in a 30°C incubator for 4 d. RT qPCR quantification of *Buchnera*/aphid DNA ratios revealed a significant decrease in the level of *Buchnera* relative to aphids not subjected to heat. Approximately 2% of the sequencing reads came from the *Buchnera* genome and were removed for separate assembly of *Buchnera* genome.

### Estimates of Genome Size

The genome size of LSR1.AC.G1 was estimated from single heads of seven asexual females by flow cytometry as described in [97] against *D. melanogaster* strain Iso-1, 1C = 175 Mb (provided by Gerald Rubin, University of California, Berkley, CA, USA).

### Sequencing and Assembly, Acyr 1.0

3.13 million Sanger sequence reads were produced on 3,730 sequencing (Applied Biosystems, Foster city, CA, USA) machines and assembled using the Atlas assembly pipeline, representing about 464 Mb of sequence and about 6.2× coverage of the (clonable) *A. pisum* genome. Two whole genome shotgun libraries, with inserts of 2–3 kb and 4–5 kb and a BAC library with insert size ∼130 kb were used to produce the data. The LSR1.AC.G1 pea aphid genome sequence is available from the NCBI with project accession ABLF01000000.

### Automated Gene Model Prediction

We took two complementary approaches to automated gene prediction. First, for high-quality evidence-based gene models, we used the NCBI evidence-based RefSeq pipeline. Second, because EST and protein homology evidence was insufficient for the RefSeq pipeline to generate a comprehensive gene model set, we supplemented the RefSeq models with a GLEAN [41] consensus set of gene models based on a collection of *ab initio* gene predictors.

The NCBI RefSeq pipeline uses a combination of homology searching with *ab initio* modeling. First cDNAs and ESTs were aligned to the genomic sequences using Splign [98] and proteins were aligned to the genomic sequences using ProSplign [99]. The best scoring coding sequence was identified for all cDNA alignments using the same scoring system used by Gnomon [100], the NCBI *ab initio* prediction tool. All cDNAs with a coding sequence scoring above a certain threshold were marked as coding cDNAs, and all others were marked as UTRs. Coding sequences that lack a translation initiation or termination signal were categorized as incomplete. Protein alignments were scored the same way, and coding sequences that did not satisfy the threshold criterion for a valid coding sequence were removed. After determining the UTR/CDS nature of each alignment, the alignments were assembled using a modification of the Maximal Transcript Alignment algorithm [101], accounting for not only exon-intron structure compatibility but also the compatibility of the reading frames. Two coding alignments were connected only if they both had open and compatible coding sequences. UTRs were connected to coding alignments only if the necessary translation initiation or termination signals were present. There were no restrictions on the connection of UTRs other than the exon-intron structure compatibility. All assembled models with a complete coding sequence, including the translation initiation and termination signals, were combined into alternatively spliced isoform groups. Incomplete or partially supported models were directed to Gnomon [100] for extension by *ab initio* prediction. Models containing a debilitating mutation such as a frameshift or nonsense mutation were categorized as either transcribed or non-transcribed pseudogenes. A subset of pseudogenes are likely to be functional genes that have errors in the Acyr_1.0 assembly and may be reclassified as protein-coding genes with subsequent improvements to the assembly and annotation. Gnomon [3] was also used to predict pure *ab initio* models in regions of the genome that lacked any cDNA, EST, or protein alignments.

Our supplemental GLEAN consensus gene model set of 36,606 was generated with input gene model sets from six different gene predictors: Augustus, FgenesH, FgenesH++, NCBI Gnomon, Maker, and NCBI RefSeq. Of these gene models, 12,251, overlapped RefSeq gene models by 100 bp or more, and in these cases, the RefSeq models were used. The final automated gene model set contains 34,604 gene models (Table 1).

### Manual Gene Annotation

Using results of computational annotation as a baseline, members of the International Aphid Genomics Consortium manually curated over 2,000 genes of biological interest. Briefly, sequences of target genes from other arthropods were utilized to blast search the RefSeq gene set, Gnomon predictions, scaffolds, and unassembled reads. Homology of putative aphid genes was verified using a combination of reciprocal blast and information garnered from phylomeDB and other phylogenetic analyses. Gene models (e.g., starts and stops, exon boundaries) were then manually refined based on available EST and full-length cDNA support, as well as alignment with homologs from other taxa. Manual curation was facilitated by an Apollo instance directly integrated with AphidBase (see below).

### AphidBase

The pea aphid assembled genome sequence data has been comprehensively scanned and annotated to highlight transcription evidence. ESTs, EST contigs, and full-length cDNAs have been mapped to the genome using SIM-4, whereas homologs in other insect genomes or Uniprot have been identified by high-throughput BLAST searches. All of the approximately 170,000 ESTs and 200 full-length cDNAs, as well as gene models generated by different programs (Augustus, RefSeq, Genscan, Maker, Snap, GeneID, Gnomon, and Fgenesh) and RefSeq and Glean gene model repertoires, were loaded into a GMOD-Chado database [102],[103] accessible at the AphidBase web portal (www.aphidbase.com; [23],[104]). Additionally, all manually curated genes are available at AphidBase.

### Species Tree Reconstruction

One hundred and ninety-seven genes with single-copy orthologs in all species included in the analyses were selected to infer a species phylogeny. Alignments performed with MUSCLE described were concatenated into a super-alignment containing 14,922 positions. The removal of positions with gaps in more than 50% of the sequences resulted in a final alignment of 90,512 positions. This alignment was used for Maximum Likelihood (ML) tree reconstruction as implemented in PhyML v2.4.4 [105], using JTT as an evolutionary model and assuming a discrete gamma-distribution model with four rate categories and invariant sites, where the gamma shape parameter and the fraction of invariant sites were estimated from the data. Bootstrap analysis was performed on the basis of 100 replicates.

### Phylome Reconstruction

We reconstructed the complete collection of phylogenetic trees, also known as the Phylome, for all *A. pisum* protein-coding genes with homologs in other sequenced insect genomes. For this we used a similar automated pipeline to that described earlier for the human genome [43]. A database was created containing the pea aphid proteome and that of 16 other species. These include 12 other insects (*Tribolium castaneum*, *Nasonia vitripennis*, *Apis mellifera* [from NCBI database], *Drosophila pseudoobscura*, *Drosophila melanogaster*, *Drosophila mojavensis*, *Drosophila yakuba* [from FlyBase], *Pediculus humanus*, *Culex pipiens* [from VectorBase], *Anopheles gambiae*, *Aedes aegypti* [from Ensembl], and *Bombyx mori* [from SILKDB]) and four outgroups (the crustacean *Daphnia pulex* [the GNOMON predicted set provided by the JGI], the nematode *Caenorhabditis elegans*, and two chordates, *Ciona intestinalis* and *Homo sapiens* [from Ensembl]). For each protein encoded in the pea aphid genome, a Smith-Waterman [106] search (e-val 10^−3^) was performed against the above mentioned proteomes. Sequences that aligned with a continuous region longer than 50% of the query sequence were selected and aligned using MUSCLE 3.6 [107] with default parameters. Gappy positions were removed using trimAl v1.0 (http://trimal.cgenomics.org), using a gap threshold of 25% and a conservation threshold of 50%. Phylogenetic trees were estimated with Neighbor Joining (NJ) trees using scoredist distances as implemented in BioNJ [108] and by ML as implemented in PhyML v2.4.4 [105], using JTT as an evolutionary model and assuming a discrete gamma-distribution model with four rate categories and invariant sites, where the gamma shape parameter and the fraction of invariant sites were estimated from the data. Support for the different partitions was computed by approximate likelihood ratio test as implemented in PhymL (aLRT) [109]. All trees and alignments have been deposited in PhylomeDB [110] (http://phylomedb.org). Additional details for this analysis can be found in [110].

### Phylogeny-Based Orthology Determination

Prediction of orthology is a fundamental step in the functional annotation of newly sequenced genomes. Reciprocal BLAST best hit is often used for genome-wide orthology detection, but phylogeny-based orthology predictions are considered more accurate, especially at large evolutionary distances or when gene duplication and loss is rampant [111]. To overcome this, orthology and paralogy relationships among *A. pisum* genes and those encoded in the other considered genomes were inferred by a phylogenetic approach that uses a previously described species-overlap algorithm [43]. This algorithm uses the level of species overlap (if there is species overlap) between the two daughter partitions of a given node to define it as a duplication or speciation (if there is no species overlap). After mapping all duplications and speciations on the phylogenetic tree of a given gene family, orthology and paralogy relationships are inferred accordingly. All orthology and paralogy predictions can be accessed through PhylomedDB [110].

### Orthology-Based Functional Annotation

A list of orthology-based transfer of functional annotations was built based on phylogeny-based orthology relationships with *Drosophila melanogaster*. Pea aphid genes with orthology relationships with annotated *D. melanogaster* genes were grouped according to the type of orthology relationship. Twelve percent (4,058) of aphid genes could be annotated based on a clear one-to-one orthology relationship with a drosophila gene. An additional 2,315 genes presented a many-to-one relationship with annotated drosophila genes and thus were tentatively annotated with the GO terms associated with the fly genes, with the caution that neo and or sub-functionalization may have occurred.

### Detection of Aphid-Specific Gene Expansions

The duplication events defined by the above mentioned species overlap algorithm that only comprised paralogs from *A. pisum* were considered lineage-specific duplications. Whenever more than one round of duplication followed an *A. pisum* speciation event (family expansion), all resulting paralogs were grouped into a single group of “in-paralogs”. Results from all the trees in the phylome were merged into a non-redundant list of in-paralogs groups, by merging groups sharing a significant fraction of their members (50%).

### Estimating the Age of Aphid-Specific Duplications

Putative pairs of paralogs were identified as pairs of genes following a reciprocal best hit criterion (RBH) within the *A. pisum* gene set; however, due to errors in the assembly process, these may comprise allelic variants found on different scaffolds (for alleles, coding sequences are expected to be extremely similar). We filtered alignments with Gblocks [112] to reduce the risk of partially non-homologous alignments and estimated the pairwise dS among genes. For comparison, the same task was performed for transcripts (not considering alternative transcripts) from *Drosophila* and honeybee genomes.

### Telomere Identification

The pea aphid has four chromosomes [113] with eight telomeres. Searches of the genome assembly for long stretches of the expected TTAGG telomeric repeat reveal several candidates, but only two are at the ends of reasonably long contigs in reasonably long scaffolds. They are ∼480 bp stretches of TTAGG repeats at the 3′ ends of 14 kb SCAFFOLD14618 (GenBank EQ125390.1) and 11 kb SCAFFOLD13146 (EQ123918.1). The remainder of these scaffolds do not encode any genes, and the subtelomeric ∼700 bp before the TTAGG repeats shows considerable sequence similarity between these two scaffolds. These are likely to be true telomeres. Unfortunately the remaining six telomeres are not assembled in scaffolds, although pieces of them might be in short single contigs. Attempts to determine their structure employed an approach similar to that utilized with the *Tribolium* genome assembly [42], involving searching of the raw reads at the Trace Archive at NCBI with a query consisting of 1000 bp of TTAGG repeats. Examination of the internal mate pairs of the first 100 such matches revealed several from the two telomeres identified above. The remainder, however, were either matches to RT domains or other regions of retrotransposons or were other simple sequence repeats. It appears, therefore, that the remaining six telomeres are rather more complicated than the two identified above, which are reminiscent of the relatively simple telomeres of the honey bee *Apis mellifera* [44]. They likely involve insertion of retrotransposons into the telomeres, much like those of the silkmoth *Bombyx mori* [45] and the red flour beetle *Tribolium castaneum* [42].

### TE Detection

TEs were identified and annotated using the “REPET” (http://urgi.versailles.inra.fr/development/repet/) pipeline, which correctly annotate nested and fragmented TEs. In the first part of the pipeline, consensus TEs were predicted *ab initio* by first searching for repeats with BLASTER for an all-by-all BLASTN [114] genome comparison and then results grouped using three clustering methods—GROUPER [115], RECON [116], and PILER [117]—with default parameters. We then built one consensus per group with the MAFFT [118] multiple sequence alignment program and classified each consensus (1) according to BLASTER matches using TBLASTX and BLASTX [114] with the entire Repbase Update databank [119] and (2) according to the presence of structural features such as terminal repeats (TIR, LTR, and polyA or SSR tails).

These TE consensus sequences representing ancestral copies of TEs subfamilies were clustered into groups for family identification using the GROUPER clustering method. Each family (i.e., group) was characterized assuming that the most populated well characterized TE category in a group of consensus sequences can define the order of the group it belongs to. Eighty-five families containing at least five TE consensus sequences were then manually curated using multiple sequences alignments, phylogenies, and Hidden Markov Models [120]. This close examination allowed us to confirm groupings and decipher specific features like chimeric TE families or subfamilies.

The pea aphid genome was annotated with all the subfamilies of TE consensus sequences using the second part of the REPET annotation pipeline. This pipeline is composed of TE detection software—BLASTER [115], RepeatMasker [121], and Censor [122]—and satellite detection software—RepeatMasker, TRF [123], and Mreps [124]. Simple repeats have been used to filter out spurious hits.

TEs often insert into other TEs fragmenting each other. A specific “long join” annotation procedure was performed, using age estimates of repeat fragments to correctly identify fragments from the same repeat. The percent identity between a fragment and its reference TE/repeat consensus can be used to estimate the age of TE fragments.

### *CpG* Analysis

CpG analysis was performed as described in [37].

### *Buchnera* Sequence

During the course of whole genome sequencing of pea aphid clones, LSR1.AC.G1, 24,947 sequence reads corresponding to the *Buchnera* genome were obtained as by-products. Using the chromatogram data of these sequences, the whole genome of *Buchnera* LSR1 was reconstructed in two distinct methods: de novo assembly using CAP3 [125] and comparative (read mapping against a reference) assembly using AMOScmp of AMOS package [126]. Results of both methods were essentially the same and the latter output was used for further analyses. Five gaps that remained after the assembly were closed by PCR reactions followed by Sanger sequencing. This *Buchnera* Whole Genome Shotgun project was deposited at DDBJ/EMBL/GenBank under the project accession ACFK00000000. The version described in this article is the first version, ACFK01000000.

### AcypiCyc Metabolism Database

A BioCyc metabolism database [55] was constructed for the pea aphid using a newly developed data management system specific for the creation and updating of Cyc databases and the BioCyc Pathway Tools. Currently, the pea aphid database, “AcypiCyc” (http://pbil.univ-lyon1.fr/software/cycads/acypicyc), utilizes the RefSeq automated annotation, complemented by three alternative annotations of the pea aphid's 34,821 proteins performed using KAAS [127]. The AcypiCyc database allows for comparison of the pea aphid database with two other BioCyc databases: SymbioCyc for *Buchnera aphidicola* APS and DromeCyc for *Drosophila melanogaster*.

## Supporting Information

Table S1 **Sanger read statistics.**
(0.04 MB DOC)

Table S2 **GC content of selected arthropod genomes.**
(0.04 MB DOC)

Table S3 **Comparison of pea aphid gene model sets to 2089 gold standard pea aphid exons from 402 genes.** bp overlap, the total number of base pairs overlapping between gold standard exons, and exons from the indicated gene model set; bp query miss, the number of bp in exons that had some overlap with the gold standard exon set but did not overlap the gold standard exon; bp target miss, the number of bp in the gold standard set that were not overlapped by the candidate gene set; any overlap, the number of gold standard exons that had 1 bp or more overlap with the gene model set in question; # correct splices, the number of gold standard exon splice sites exactly predicted by the gene model set in question; # within 6 bp, the number of splice site within 6bp, not including those exactly predicted.
(0.04 MB DOC)

Table S4 **Arthropod gene structure statistics.** Genome size value in parentheses is total gene-containing sequence (i.e., excluding heterochromatin, scaffolds without genes, etc.). No. of genes is from the gene set examined, not necessarily the official gene set for new genomes. Gene density is calculated as the sum of coding exon bases/total gene-containing genome bases. Gene length is the span including introns and UTR. CDS size is the coding sequence length without introns or UTRs. Exons/gene and Exon size are count and size of coding exons. Sizes are given as mean in bp except for Intron size. Intergenic size is measured from distance between adjacent genes. These statistics have a standard deviation close to the mean, but Intergenic size has a much larger variance. ^1^ Gene part sizes and exons/gene are measured with EST-validated gene models for these noted genomes. Others are measured from reference database gene feature data. ^2^ Exon size distribution for Drosophila is strongly bimodal; one-exon genes average twice the size of multi-exon genes (830 bp versus 470 bp/exon). Other species show unimodal distribution of exon sizes. ^3^ Intron size is non-normally distributed. Intron size lists the primary and secondary peaks, mean, and the percent of introns larger than exons. It has a narrow, high peak frequency at the indicated (median) value. Fruitfly and nematode have a secondary peak at about 400 bp; mouse reverses this with its secondary peak at 90 bp. Daphnia appears to have no secondary intron size peak. ^4^ UTR size is an overestimate, as it is measured only where exons extend past coding sequence, and misses true cases of zero length UTRs. Genome sequences used: Aphid, *Acyr. pisum* (acyr1); Beetle, *Tribolium castenatum* (tcas3); Bee, *Apis mellifera* (ncbi1); Daphnia, *Daphnia pulex* (daphx1); Fruitfly, *Drosophila melanogaster* (fb5.5); Mosquito, *Culex pipens* (cpip12); Mouse, *Mus musculus* (mgi3); Wasp, *Nasonia vitripennis* (nvit1); Worm, *Caen. elegans* (wb167).
(0.05 MB DOC)

Table S5 **Diagnostic PCR to check the presence/absence of scaffolds that appeared to be bacterial contaminants.** Among 642 PPPs located in scaffolds that appeared to be of bacterial contaminants, 46 were portions of 42 RefSeq aphid gene models. We performed diagnostic PCRs to check the presence/absence of these genes/scaffolds in the *A. piusm* genome. Specific primers were designed for each unique target gene. Each 30 µL PCR reaction contained 0.5 µM each primer, 0.2 µM dNTPs, 10 ng template, and 2.5 U AmpliTaq (Applied Biosystems) in 1× AmpliTaq buffer. Parameters for PCRs were: 94°C for 30 s, followed by 35 cycles of 94°C for 15 s, 50°C for 30 s, 72°C for 1.5 min, 72°C for 10 min and, 4°C hold. *LdcA1* was used as a positive control. PCR primers for *LdcA1* were Ap_ldcA_482F (5′-TATGATACCGTACCTGGAGGCGTT-3′) and Ap_ldcA_1127R′ (5′-GTTTTAATCACGCAGCACATGGG-3′). None of the target DNA sequences were amplified by PCR, verifying the absence of these scaffolds in the aphid genome.
(0.12 MB DOC)

Table S6 **Distribution of reactions in the AcypiCyc database across the six top-level categories identified by the Enzyme Commission (EC).** Included in this table are all reactions in the AcypiCyc database that have been assigned either full or partial EC numbers.
(0.04 MB DOC)

## Abbreviations

AMP: antimicrobial peptide
CBD: chitin-binding domain
CCEs: carboxyl/choline esterases
CSPs: chemosensory proteins
GPCR: G protein-coupled receptor
GRs: gustatory receptors
GSTs: glutathione *S-*transferases
JH: juvenile hormone
MFS: major facilitator superfamily
ML: Maximum Likelihood
NJ: Neighbor Joining
OBPs: odorant-binding proteins
Ors: odorant receptors
P450s: P450 monooxygenases
PGRPs: peptidoglycan recognition proteins
RBH: reciprocal best hit
RISC: RNA Induced Silencing Complex
TE: transposable element
