# Correction: Genome Sequence of the Pea Aphid *Acyrthosiphon pisum*

**DOI:** 10.1371/journal.pbio.3000029

**Published:** 2018-09-13

**Authors:** The International Aphid Genomics Consortium

The Acknowledgments contain an error. J. Spencer Johnston should be listed as a member of The International Aphid Genomics Consortium (IAGC).

J. Spencer Johnston is affiliated with the Department of Entomology, Texas A&M University, College Station, Texas, United States of America.

The Acknowledgements should read:

**The members of The International Aphid Genomics Consortium (IAGC) are as follows: Sequencing leadership:** Stephen Richards, Richard A. Gibbs, **Project Leadership:** Nicole M. Gerardo, Nancy Moran, Atsushi Nakabachi, Stephen Richards, David Stern, Denis Tagu, Alex C. C. Wilson; **DNA sequence and global analysis: DNA sequencing: Sequence Production:** Donna Muzny, Christie Kovar, Andy Cree, Joseph Chacko, Mimi N. Chandrabose, Marvin Diep Dao, Huyen H. Dinh, Ramatu Ayiesha Gabisi, Sandra Hines, Jennifer Hume, Shalini N. Jhangian, Vandita Joshi, Lora R. Lewis, Yih-shin Liu, John Lopez, Margaret B. Morgan, Ngoc Bich Nguyen, Geoffrey O. Okwuonu, San Juana Ruiz, Jireh Santibanez, Rita A. Wright; **Sequence Production Informatics:** Gerald R. Fowler, Matthew E. Hitchens, Ryan J. Lozado, Charles Moen, David Steffen, James T. Warren, Jingkun Zhang; **Sequence Library Production:** Lynne V. Nazareth, Dean Chavez, Clay Davis, Sandra L. Lee, Bella Mayurkumar Patel, Ling-Ling Pu, Stephanie N. Bell, Angela Jolivet Johnson, Selina Vattathil, Rex L. Williams Jr.; **Full length ESTs:** Shuji Shigenobu, David Stern, Stephen Richards, Phat M. Dang, Mizue Morioka, Takema Fukatsu, Toshiaki Kudo, Shin-ya Miyagishima, Atsushi Nakabachi; **Genome size:** J. Spencer Johnston; **Genome Assembly:** Huaiyang Jiang, Stephen Richards, Kim C. Worley; **AphidBase and bioinformatics resources:** Fabrice Legeai, Jean-Pierre Gauthier, Olivier Collin, Shuji Shigenobu, Denis Tagu; **Gene prediction and consensus gene set:** Fabrice Legeai, Lan Zhang, Jean-Pierre Gauthier, Shuji Shigenobu, Denis Tagu, Stephen Richards, Hsiu-Chuan Chen, Olga Ermolaeva, Wratko Hlavina, Yuri Kapustin, Boris Kiryutin, Paul Kitts, Donna Maglott, Terence Murphy, Kim Pruitt, Victor Sapojnikov, Alexandre Souvorov, Françoise Thibaud-Nissen, Francisco Câmara, Roderic Guigó, Mario Stanke, Victor Solovyev, Peter Kosarev, Don Gilbert; **Phylogenomic Analyses:** Toni Gabaldón, Jaime Huerta-Cepas, Marina Marcet-Houben, Miguel Pignatelli, Don Gilbert, Andrés Moya; **Gene duplications:** Claude Rispe, Morgane Ollivier, Fabrice Legeai, Denis Tagu; **Transposable elements:** Hadi Quesneville, Emmanuelle Permal, Andrés Moya, Carlos Llorens, Ricardo Futami, Alex C. C. Wilson, Dale Hedges; **Telomeres:** Hugh M. Robertson; **U12-Introns and Seleno-Proteins:** Tyler Alioto, Marco Mariotti, Roderic Guigó; **Symbiosis: Bacterial and mitochondrial genes in the aphid genome:** Naruo Nikoh, John P. McCutcheon, Miguel Pignatelli, Gaelen Burke, Nicole M. Gerardo, Alexandra Kamins, Amparo Latorre, Andrés Moya, Toshiaki Kudo, Shin-ya Miyagishima, Nancy A. Moran, Atsushi Nakabachi; **Metabolism:** Peter Ashton, Federica Calevro, Hubert Charles, Stefano Colella, Angela Douglas, Georg Jander, Derek H. Jones, Gérard Febvay, Lars G. Kamphuis, Philip F. Kushlan, Sandy Macdonald, John Ramsey, Julia Schwartz, Stuart Seah, Gavin Thomas, Augusto Vellozo, Alex C. C. Wilson; **Comparative genomics of Buchnera:** Shuji Shigenobu, Stephen Richards, Nancy Moran, Shin-ya Miyagishima, Atsushi Nakabachi; **Genome analysis of Regiella insecticola:** Bodil Cass, Patrick Degnan, Bonnie Hurwitz, Teresa Leonardo, Ryuichi Koga, Nancy Moran, Stephen Richards, David Stern; **Stress and immunity group:** Boran Altincicek, Caroline Anselme, Hagop Atamian, Seth M. Barribeau, Martin de Vos, Elizabeth J. Duncan, Jay Evans, Toni Gabaldon, Nicole M. Gerardo, Murad Ghanim, Abdelaziz Heddi, Isgouhi Kaloshian, Amparo Latorre, Carole Vincent-Monegat, Andrés Moya, Atsushi Nakabachi, Ben J. Parker, Vicente Pérez-Brocal, Miguel Pignatelli, Yvan Rahbé, John Ramsey, Chelsea J. Spragg, Javier Tamames, Daniel Tamarit, Cecilia Tamborindeguy, Andreas Vilcinskas; **Development group:** Shuji Shigenobu, Ryan D. Bickel, Jennifer A. Brisson, Thomas Butts, Chun-che Chang, Olivier Christiaens, Gregory K. Davis, Elizabeth Duncan, David Ferrier, Masatoshi Iga, Ralf Janssen, Hsiao-Ling Lu, Alistair McGregor, Toru Miura, Guy Smagghe, James Smith, Maurijn van der Zee, Rodrigo Velarde, Megan Wilson, Peter Dearden, David Stern; **Germ line group:** Chun-che Chang, Hsiao-Ling Lu, Ryan D. Bickel, Shuji Shigenobu, Gregory K. Davis; **Epigenetics and Methylation:** Jennifer A. Brisson, Owain R. Edwards, Karl Gordon, Roland S. Hilgarth, Stanley Dean Rider Jr., Hugh M. Robertson, Dayalan Srinivasan, Thomas K. Walsh; **Wing development:** Jennifer A. Brisson, Asano Ishikawa, Toru Miura; **JH-related:** Toru Miura, Jennifer A. Brisson, Asano Ishikawa, Stéphanie Jaubert-Possamai, Denis Tagu, Thomas K. Walsh; **Mitosis, meiosis and cell cycle:** Dayalan Srinivasan, Brian Fenton, Stéphanie Jaubert-Possamai; **Sex determination:** Wenting Huang, Derek H. Jones, Alex C. C. Wilson; **MicroRNA and phenotypic plasticity:** Fabrice Legeai, Thomas K. Walsh, Guillaume Rizk, Owain R. Edwards, Karl Gordon, Dominique Lavenier, Jacques Nicolas, Denis Tagu, Stéphanie Jaubert-Possamai, Claude Rispe; **Aphid Plant Interactions: Chemoreceptors:** Carole Smadja, Hugh M. Robertson; **Odorant-Binding Proteins:** Jing-Jiang Zhou, Filipe G. Vieira, Carole Smadja, Xiao-Li He, Renhu Liu, Julio Rozas, Linda M. Field; **Detoxification enzymes:** Stanley Dean Rider Jr., John Ramsey, Karl Gordon, Thomas K. Walsh, Martin de Vos, Georg Jander; **Salivary glands:** Peter D. Ashton, Peter Campbell, James C. Carolan, Angela E. Douglas, Owain R. Edwards, Carol I. J. Fitzroy, Lars G. Kamphuis, Karen T. Reardon, Gerald R. Reeck, Karam Singh, Thomas L. Wilkinson; **Neuropeptides:** Jurgen Huybrechts, Mohatmed Abdel-latief, Alain Robichon, Jan A. Veenstra, Frank Hauser, Giuseppe Cazzamali, Martina Schneider, Michael Williamson, Elisabeth Stafflinger, Karina K. Hansen, Cornelis J. P. Grimmelikhuijzen, Denis Tagu; **Transporters:** Daniel R.G Price, Marina Caillaud, Eric van Fleet, Qinghu Ren, Yvan Rahbé, Angela E. Douglas, John A. Gatehouse; **Virus transmission and transcytosis group:** Véronique Brault, Baptiste Monsion, Marina Caillaud, Eric Van Fleet, Jason Diaz, Laura Hunnicutt, Atsushi Nakabachi, Ho-Jong Ju, Cecilia Tamborindeguy, Ximo Pechuan, José Aguilar, Daniel Tamarit; Carlos Llorens, Andres Moya; **Dynamins:** Atsushi Nakabachi, Shin-ya Miyagishima; **Circadian rhythms group:** Teresa Cortés, Benjamín Ortiz-Rivas, David Martínez-Torres; **Cuticular proteins:** Claude Rispe, Aviv Dombrovsky, Stéphanie Jaubert-Possamai, Denis Tagu; **Chitinase-like proteins:** Atsushi Nakabachi, Shuji Shigenobu, Shin-ya Miyagishima; **Ion Channels:** Richard P. Dale, Thomas K. Walsh, Cecilia Tamborindeguy, T. G. Emyr Davies, Linda M. Field, Martin S. Williamson, Andrew Jones, David Sattelle, Sally Williamson, Adrian Wolstenholme; **Protease genes:** Peter Campbell, James C. Carolan, Owain R. Edwards, Karl Gordon, Carlos Llorens, Andres Moya, Miguel Pignatelli, Yvan Rahbé, Claude Rispe, Gerald R. Reeck; **AcypiCyc:** Augusto Vellozo, Stefano Colella, Ludovic Cottret, Gérard Febvay, Federica Calevro, Yvan Rahbé, Angela Douglas, Marie France Sagot, Hubert Charles; **Ribosomal Proteins:** Claude Rispe, David G. Heckel, Wayne Hunter.
