# Supplementary material for: Genome Sequence of the Pea Aphid Acyrthosiphon pisum
Source: PLoS Biol. 2010 Feb 23;8(2):e1000313. doi: 10.1371/journal.pbio.1000313 (PMC2826372; doi:10.1371/journal.pbio.1000313)
Supplement: Table S1 — Sanger read statistics. (0.04 MB DOC) [file pbio.1000313.s001.doc]

***Acyrthosiphon pisum***

***The International Aphid Genomics Consortium***

Table S1. Sanger Read Statistics.

| **Insert Size** | **Raw** | **Passed QC** | **Assembled reads** | **Clone** |
| --- | --- | --- | --- | --- |
| 2-5 kb | 4,325,313 | 3,955,990 | 3,044,414 | plasmid |
| 35 kb | 24,673 | 8,158 | 5,294 | fosmid |
| 110-130 kb | 56,246 | 45,140 | 2,286 | BAC |
| TOTAL | 4,406,232 | 4,009,288 | 3,051,994 |  |
