# Supplementary material for: Genome Sequence of the Pea Aphid Acyrthosiphon pisum
Source: PLoS Biol. 2010 Feb 23;8(2):e1000313. doi: 10.1371/journal.pbio.1000313 (PMC2826372; doi:10.1371/journal.pbio.1000313)
Supplement: Table S2 — GC content of selected arthropod genomes. (0.04 MB DOC) [file pbio.1000313.s002.doc]

***Acyrthosiphon pisum***

***The International Aphid Genomics Consortium***

Table S2. GC content of selected arthropod genomes.

| **Size_Mb** | **GC_Pct** | **NN_Pct** | **Genome_assembly** |
| --- | --- | --- | --- |
| **464** | **29.6** | **4** | **Aphid Acyr20071212-genome** |
| 176 | 33.9 | 30 | Tribolium chromosomes |
| 217 | 34.8 | 16 | Apis amel4-chromosomes |
| 100 | 35.4 | 0 | Nematode celegans-dna-WS167 |
| **36** | **38.0** | **Na** | **aphid_transcripts** |
| 20 | 39.1 | Na | amel4-transcripts |
| 294 | 41.7 | 19 | Nasonia nvi_ref_chrUn |
| 227 | 41.5 | 27 | Daphnia dpulex_jgi060905 |
| 129 | 42.3 | 4.6 | dmel_caf060213 |
| 1765 | 45.2 | 21 | Ixodes iscapularis.IscaW1 |
| 33 | 45.6 | Na | nasonia_transcripts |
| 21 | 46.1 | Na | tribolium_transcripts |
| 38 | 47.2 | Na | dpulex_transcripts |
| Size_Mb = assembly size in megabases | | | |
| GC_Pct -= percent G+C/(G+C+A+T) | | | |
| NN_Pct = percent NNN gaps of total assembly | | | |
