# Supplementary material for: Genome Sequence of the Pea Aphid Acyrthosiphon pisum
Source: PLoS Biol. 2010 Feb 23;8(2):e1000313. doi: 10.1371/journal.pbio.1000313 (PMC2826372; doi:10.1371/journal.pbio.1000313)
Supplement: Table S3 — Comparison of pea aphid gene model sets to 2089 gold standard pea aphid exons from 402 genes. bp overlap, the total number of base pairs overlapping between gold standard exons, and exons from the indicated gene model set; bp query miss, the number of bp in exons that had some overlap with the gold standard exon set but did not overlap the gold standard exon; bp target miss, the number of bp in the gold standard set that were not overlapped by the candidate gene set; any overlap, the number of gold standard exons that had 1 bp or more overlap with the gene model set in question; # correct splices, the number of gold standard exon splice sites exactly predicted by the gene model set in question; # within 6 bp, the number of splice site within 6bp, not including those exactly predicted. (0.04 MB DOC) [file pbio.1000313.s003.doc]

***Acyrthosiphon pisum***

***The International Aphid Genomics Consortium***

**Table S3. Comparison of Pea Aphid Gene Model Sets to 2089 Gold Standard Pea Aphid Exons from 402 Genes.**

|  | GLEAN | RefSeq | Fgenesh++ | Augustus | Maker | fgenesh | Gnomon | geneid | genscan |
| --- | --- | --- | --- | --- | --- | --- | --- | --- | --- |
| bp overlap | 388,305 | 378,725 | 382,233 | 356,806 | 350,042 | 383,882 | 387,689 | 376,499 | 280,470 |
| Bp query miss | 10,017 | 7,558 | 5,255 | 17,089 | 20,831 | 3,359 | 18,594 | 4,322 | 11,516 |
| Bp target miss | 3,323 | 137,272 | 4,279 | 3,927 | 5,890 | 5,089 | 4,041 | 6,732 | 7,935 |
| # any overlap | 2,029 | 1,994 | 1,964 | 1,904 | 1,898 | 1,964 | 2,053 | 1,906 | 1,268 |
| #correct splices | 3,902 | 3,311 | 3,726 | 3,657 | 3,344 | 3,750 | 3,922 | 3,580 | 2,208 |
| # within 6bp | 50 | 62 | 51 | 39 | 106 | 53 | 53 | 66 | 59 |

bp overlap: indicates the total number of base pairs overlapping between gold standard exons, and exons from the indicated gene model set.

bp query miss: is the number of bp in exons that had some overlap with the gold standard exon set, but did not overlap the gold standard exon.

bp target miss: is the number of bp in the gold standard set that had were not overlapped by the candidate gene set.

any overlap: The number of gold standard exons that had 1bp or more overlap with the gene model set in question.

# correct splices: The number of gold standard exon splice sites exactly predicted by the gene model set in question.

# within 6bp: The number of splice site within 6bp, not including those exactly predicted.
