# Supplementary material for: Genome Sequence of the Pea Aphid Acyrthosiphon pisum
Source: PLoS Biol. 2010 Feb 23;8(2):e1000313. doi: 10.1371/journal.pbio.1000313 (PMC2826372; doi:10.1371/journal.pbio.1000313)
Supplement: Table S4 — Arthropod gene structure statistics. Genome size value in parentheses is total gene-containing sequence (i.e., excluding heterochromatin, scaffolds without genes, etc.). No. of genes is from the gene set examined, not necessarily the official gene set for new genomes. Gene density is calculated as the sum of coding exon bases/total gene-containing genome bases. Gene length is the span including introns and UTR. CDS size is the coding sequence length without introns or UTRs. Exons/gene and Exon size are count and size of coding exons. Sizes are given as mean in bp except for Intron size. Intergenic size is measured from distance between adjacent genes. These statistics have a standard deviation close to the mean, but Intergenic size has a much larger variance. 1 Gene part sizes and exons/gene are measured with EST-validated gene models for these noted genomes. Others are measured from reference database gene feature data. 2 Exon size distribution for Drosophila is strongly bimodal; one-exon genes average twice the size of multi-exon genes (830 bp versus 470 bp/exon). Other species show unimodal distribution of exon sizes. 3 Intron size is non-normally distributed. Intron size lists the primary and secondary peaks, mean, and the percent of introns larger than exons. It has a narrow, high peak frequency at the indicated (median) value. Fruitfly and nematode have a secondary peak at about 400 bp; mouse reverses this with its secondary peak at 90 bp. Daphnia appears to have no secondary intron size peak. 4 UTR size is an overestimate, as it is measured only where exons extend past coding sequence, and misses true cases of zero length UTRs. Genome sequences used: Aphid, Acyr. pisum (acyr1); Beetle, Tribolium castenatum (tcas3); Bee, Apis mellifera (ncbi1); Daphnia, Daphnia pulex (daphx1); Fruitfly, Drosophila melanogaster (fb5.5); Mosquito, Culex pipens (cpip12); Mouse, Mus musculus (mgi3); Wasp, Nasonia vitripennis (nvit1); Worm, Caen. elegans (wb167). (0.05 MB DOC) [file pbio.1000313.s004.doc]

***Acyrthosiphon pisum***

***The International Aphid Genomics Consortium***

**Table S4. Arthropod gene structure statistics**

|  | **Daphnia**^1^ | **Aphid**^1^ | **Bee** | **Wasp**^1^ | **Beetle** | **Mosquito** | **Fruitfly**^1^ | **Mouse** | **Worm** |
| --- | --- | --- | --- | --- | --- | --- | --- | --- | --- |
| Genome size (Mbp) | 200  (175) | 460  (350) | 220  (200) | 290  (250) | 180  (177) | 580  (400) | 180  (120) | 3,450  (2,600) | 100  (100) |
| Gene  number | 32,000 | 32,800 | 17,000 | 27,300 | 16,400 | 18,900 | 13,700 | 20,600 | 20,100 |
| Gene density | 0.175 | 0.063 | 0.040 | 0.120 | 0.100 | 0.055 | 0.168 | 0.015 | 0.250 |
| Gene length | 2,200 | 4,500 | 3,900 | 2,900 | 3,700 | 3,700 | 3,200 | 8,300 | 3,000 |
| CDS size | 1,320 | 1,200 | 1,590 | 1,510 | 1,370 | 1,280 | 1,650 | 1,820 | 1,300 |
| Exons/gene | 6.5 | 6.3 | 6.8 | 6.0 | 4.4 | 3.5 | 4.3 | 6.3 | 6.0 |
| Exon size ^2^ | 210 | 200 | 240 | 260 | 310 | 360 | 410 | 280 | 200 |
| Intron size^3^  Mean Intr > Exon | 72  170  10% | 71/640  790  41% | 79/290  770  36% | 79/310  430  24% | 57/1200  1000  31% | 65/1100  1600  37% | 69/400  660  27% | 1200/90  2800  85% | 65/400  290  33% |
| UTR size ^4^ | 370 | 490 | 340 | 680 | -- | 190 | 750 | -- | 260 |
| Intergenic size | 4,000 | 6,500 | 9,200 | -- | 7,900 | 17,500 | 4,700 | 68,000 | 2,400 |

Updated 2009/01/19. Compiled by D.Gilbert, gilbertd@indiana.edu.

**Genome size** value in parentheses is total gene-containing sequence (i.e. excluding heterochromatin, scaffolds without genes, etc.). **No. of genes** is from the gene set examined, not necessarily the official gene set for new genomes. **Gene density** is calculated as the sum of coding exon bases / total gene-containing genome bases.  **Gene length** is the span including introns and UTR. **CDS size** is the coding sequence length without introns or UTRs. **Exons/gene** and **Exon size** are count and size of coding exons. Sizes are given as mean in bp except for Intron size. **Intergenic size** is measured from distance between adjacent genes. These statistics have a standard deviation close to the mean, but Intergenic size has a much larger variance.

^1^ Gene part sizes and exons/gene are measured with EST-validated gene models for these noted genomes. Others are measured from reference database gene feature data.

^2^ **Exon size** distribution for *Drosophila* is strongly bimodal; one-exon genes average twice the size of multi-exon genes (830 bp versus 470 bp/exon). Other species show unimodal distribution of exon sizes.

^3^ **Intron size** is non-normally distributed. Intron size lists the primary and secondary peaks, mean and the percent of introns larger than exons. It has a narrow, high peak frequency at the indicated (median) value. Fruitfly and nematode have a secondary peak at about 400 bp, mouse reverses this with its secondary peak at 90 bp. Daphnia appears to have no secondary intron size peak.

^4^ **UTR size** is an over-estimate, as it is measured only where exons extend past coding sequence, and misses true cases of zero length UTRs.

Genome sequences used: Aphid = *Acyr. pisum* (acyr1); Beetle = *Tribolium castenatum* (tcas3); Bee = *Apis mellifera* (ncbi1); Daphnia = *Daphnia pulex* (daphx1); Fruitfly = *Drosophila melanogaster* (fb5.5); Mosquito = *Culex pipens* (cpip12); Mouse = *Mus musculus* (mgi3); Wasp = *Nasonia vitripennis* (nvit1); Worm = *Caen. elegans* (wb167);
