# Supplementary material for: Genome Sequence of the Pea Aphid Acyrthosiphon pisum
Source: PLoS Biol. 2010 Feb 23;8(2):e1000313. doi: 10.1371/journal.pbio.1000313 (PMC2826372; doi:10.1371/journal.pbio.1000313)
Supplement: Table S5 — Diagnostic PCR to check the presence/absence of scaffolds that appeared to be bacterial contaminants. Among 642 PPPs located in scaffolds that appeared to be of bacterial contaminants, 46 were portions of 42 RefSeq aphid gene models. We performed diagnostic PCRs to check the presence/absence of these genes/scaffolds in the A. piusm genome. Specific primers were designed for each unique target gene. Each 30 µL PCR reaction contained 0.5 µM each primer, 0.2 µM dNTPs, 10 ng template, and 2.5 U AmpliTaq (Applied Biosystems) in 1× AmpliTaq buffer. Parameters for PCRs were: 94°C for 30 s, followed by 35 cycles of 94°C for 15 s, 50°C for 30 s, 72°C for 1.5 min, 72°C for 10 min and, 4°C hold. LdcA1 was used as a positive control. PCR primers for LdcA1 were Ap_ldcA_482F (5′-TATGATACCGTACCTGGAGGCGTT-3′) and Ap_ldcA_1127R′ (5′-GTTTTAATCACGCAGCACATGGG-3′). None of the target DNA sequences were amplified by PCR, verifying the absence of these scaffolds in the aphid genome. (0.12 MB DOC) [file pbio.1000313.s005.doc]

***Acyrthosiphon pisum***

***The International Aphid Genomics Consortium***

**Table S5. RefSeq gene models in scaffolds demonstrated to be bacterial contaminants using PCR experiments.**

| Protein | LOC_ID | ACYPI_ID | GenBank ID | Length | Forward Primer 5'-3' | Reverse Primer 5'-3' | PCR |
| --- | --- | --- | --- | --- | --- | --- | --- |
| XP_001951841.1 | LOC100158847 | ACYPI000269 | EQ111627 | 2663 | CTCGCGGATATGATGTGATG | CTCCTGAATAATGCCGGTGT | - |
| XP_001948966.1 | LOC100168149 | ACYPI008875 | EQ111762 | 1935 | CTGCACGTAGCACACCAAGT | GCATGAAGTTTCTGGCATCA | - |
| XP_001949015.1 | LOC100166112 | ACYPI007013 | EQ111763 | 1935 | CTGCACGTAGCACACCAAGT | GCATGAAGTTTCTGGCATCA | - |
| XP_001950629.1 | LOC100162046 | ACYPI003227 | EQ112016 | 4663 | CAACGCATCTGTCGCTTAAA | CGTGACAGCATCAAGGCTTA | - |
| XP_001951960.1 | LOC100169056 | ACYPI009709 | EQ112969 | 4257 | GCAAACCCAGGTGAAACAGT | TTTCGTACCATCTTGCATCG | - |
| XP_001952695.1 | LOC100162865 | ACYPI003987 | EQ113051 | 2112 | TTGGTGAAGTGTGGAGCAAG | CTCTGCTACGCCCTTGTAGG | - |
| XP_001946386.1 | LOC100161353 | ACYPI002582 | EQ113148 | 1485 | TTCTCAGCGCAAAGTGCTTA | CACATTTTCAGCGTCTGTGG | - |
| XP_001950066.1 | LOC100167452 | ACYPI008249 | EQ113955 | 5281 | GGTATCACTGAACCCGCAGT | CACCCCATAAAAATGCATCC | - |
| XP_001952752.1 | LOC100165450 | ACYPI006401 | EQ114168 | 2219 | TCAGGTTGTCCACGTAGCTG | CGGACGACCGTCTTGTTTAT | - |
| XP_001944029.1 | LOC100159091 | ACYPI000494 | EQ114489 | 5331 | TGGCTTAACTGGGGTAATCG | ATGACTTTTGCGGTCAAACC | - |
| XP_001951051.1 | LOC100159792 | ACYPI001139 | EQ114632 | 1828 | ACGATGGTGGAAATGGGTAA | TAGATTCGCCGAGCTCATTT | - |
| XP_001951397.1 | LOC100166168 | ACYPI007063 | EQ115931 | 7856 | GGGAATCATCAACCATCACC | GGTTTCACGCACGAATACCT | - |
| XP_001952057.1 | LOC100167626 | ACYPI008405 | EQ116468 | 4923 | GGGCACCTGAATAATGCCTA | TGACTCATGTTTCGCTCCAG | - |
| XP_001942694.1 | LOC100162592 | ACYPI003738 | EQ116471 | 4638 | CCGAATCCGGTTATGAATTG | GGAGCCGATGTTTACGATGT | - |
| XP_001950577.1 | LOC100163942 | ACYPI004991 | EQ116650 | 1545 | TGCGGCATAACTGCATAAAG | CCGGTTATCACACTGCTGAA | - |
| XP_001947334.1 | LOC100165848 | ACYPI006770 | EQ117052 | 1832 | ATCTACAACGTCTGCAACTACAC | GTAGTTCAAAGTGACTGACATCC | - |
| XP_001948611.1 | LOC100167171 | ACYPI007985 | EQ117084 | 1074 | CAATGTGACCTTTACCTATCC | GCTTCATCAAGGATCAGAATC | - |
| XP_001944417.1 | LOC100158836 | ACYPI000260 | EQ117551 | 4139 | CTGAAAGGCGAGGTGTATCTG | ATGCTGCTGATTCAACTCCTGC | - |
| XP_001951375.1 | LOC100169205 | ACYPI009845 | EQ118618 | 1466 | TTCCCAGATGTTCAAGTAAGAG | GTCATCTATCGTACCGTCTTC | - |
| XP_001952505.1 | LOC100165121 | ACYPI006086 | EQ118725 | 1244 | ATGTTTTCTCCTCACTCCTG | GGCAGGCATTTATCAGAATAGTG | - |
| XP_001950427.1 | LOC100165957 | ACYPI006868 | EQ119027 | 1947 | TGAAGAAGAAGGTGGAGAAAG | GTTCTTGTAGGTGataTCGCCGC | - |
| XP_001952563.1 | LOC100168647 | ACYPI009328 | EQ119168 | 823 | AGCGTCACGTAAACGCATTG | TCAGATCGGCGATGTATTGG | - |
| XP_001952789.1 | LOC100167282 | ACYPI008091 | EQ119243 | 1701 | ACTCGTCGCCTTGTTTAACTG | ATGATGGGTGAAGGATTCGG | - |
| XP_001947797.1 | LOC100169451 | ACYPI010067 | EQ119552 | 1384 | GCACCACGGCTTTTACTCCTTTG | CGTTCCACATCATCAGCAGTTCC | - |
| XP_001950910.1 | LOC100161907 | ACYPI003095 | EQ119651 | 1557 | CCGAGGTTGGAGGTCATCAGG | CAGATGGTGAAGGTGGACGAGC | - |
| XP_001950457.1 | LOC100168187 | ACYPI008913 | EQ119988 | 4546 | GCTGTTACCGAAAATCACGCCAC | GGATCGCACCAGTCTTCAAGC | - |
| XP_001951934.1 | LOC100159928 | ACYPI001267 | EQ122070 | 1316 | GAAACAGCATCAGGCAGAAGTG | GTTAGCAGCAAGGTCTCAAGGTG | - |
| XP_001946969.1 | LOC100166329 | ACYPI007211 | EQ123444 | 5168 | GGCTTCTACCTCTTGTTTCTGG | GGATGGTTGGATTAGCAGTTGTC | - |
| XP_001946787.1 | LOC100161534 | ACYPI002750 | EQ123444 | 5168 | not tested as the same scaffold as above | | N/A |
| XP_001946904.1 | LOC100168391 | ACYPI009096 | EQ123444 | 5168 | not tested as the same scaffold as above | | N/A |
| XP_001952707.1 | LOC100167696 | ACYPI008466 | EQ123665 | 1578 | TGCTCTCGTAACAGGCTCAGG | ACGGAAGGCGTCAATGGCAGC | - |
| XP_001948056.1 | LOC100162927 | ACYPI004046 | EQ124982 | 4892 | CACAGCGTTACTCCAGTCCTCC | CTATCACCACCTGCTCACCATC | - |
| XP_001943310.1 | LOC100162058 | ACYPI003238 | EQ125735 | 1697 | TGTGGCTTATGCGCTGTTAG | GTGCCGGAATATGATCGACT | - |
| XP_001946284.1 | LOC100162118 | ACYPI003292 | EQ126368 | 1876 | CTGAAAACACGGTTGCAAGA | AATTCCATGCATCCCCATTA | - |
| XP_001952731.1 | LOC100168337 | ACYPI009047 | EQ126969 | 1508 | TGCTTGGGGGATTTTACAAG | GATCGCCATTTCTTGACGTT | - |
| XP_001951869.1 | LOC100162933 | ACYPI004052 | EQ127266 | 1095 | TAACTGCGAGTGGTGGATCA | TCTTTCTTCATCGCTTGCTG | - |
| XP_001950108.1 | LOC100162986 | ACYPI004104 | EQ127803 | 1205 | GCATATGCAGATTGGGAAGTC | TAGCTGCAGGTACGCCTGTT | - |
| XP_001942584.1 | LOC100159538 | ACYPI000898 | EQ130335 | 816 | CAGTATCAGGATCAAGAGTCACG | ATAACCTCGACCGCATGAAG | - |
| XP_001942848.1 | LOC100160239 | ACYPI001553 | EQ131142 | 726 | CCGAGAACGGGGTCTG | TACAGAAGGTGCGCATGAAG | - |
| XP_001942792.1 | LOC100162287 | ACYPI003447 | EQ131135 | 727 | GCGCTCAAAATACTGCGTTA | GCGCTCAAAATACTGCGTTA | - |
| XP_001943059.1 | LOC100162855 | ACYPI003978 | EQ131157 | 725 | not tested, assembled with 19563 |  | N/A |
| XP_001942899.1 | LOC100169109 | ACYPI009756 | EQ131190 | 722 | not tested, assembled with 20363 |  | N/A |
| XP_001942584.1 | LOC100159538 | ACYPI000898 | EQ132427 | 623 | not tested, assembled with 19563 |  | N/A |

**Table S5 Diagnostic PCR to check the presence/absence of scaffolds that appeared to be bacterial contaminants.**

Among 642 PPPs located in scaffolds that appeared to be of bacterial contaminants, 46 were portions of 42 RefSeq aphid gene models. We performed diagnostic PCRs to check the presence/absence of these genes/scaffolds in the *A. piusm* genome. Specific primers were designed for each unique target gene. Each 30μL PCR reaction contained 0.5 μM each primer, 0.2 μM dNTPs, 10 ng template, and 2.5 U AmpliTaq (Applied Biosystems) in 1X AmpliTaq buffer. Parameters for PCRs were: 94°C for 30 seconds; followed by 35 cycles of 94°C for 15 sec, 50°C for 30 sec, 72°C for 1.5 min; 72°C for 10 min; 4°C hold. *LdcA1* was used as a positive control. PCR primers for *LdcA1* were Ap_ldcA_482F (5’- TATGATACCGTACCTGGAGGCGTT –3’) and Ap_ldcA_1127R’ (5’- GTTTTAATCACGCAGCACATGGG –3’). None of the target DNA sequences were amplified by PCR, verifying the absence of these scaffolds in the aphid genome.
