# Supplementary material for: Genome Sequence of the Pea Aphid Acyrthosiphon pisum
Source: PLoS Biol. 2010 Feb 23;8(2):e1000313. doi: 10.1371/journal.pbio.1000313 (PMC2826372; doi:10.1371/journal.pbio.1000313)
Supplement: Table S6 — Distribution of reactions in the AcypiCyc database across the six top-level categories identified by the Enzyme Commission (EC). Included in this table are all reactions in the AcypiCyc database that have been assigned either full or partial EC numbers. (0.04 MB DOC) [file pbio.1000313.s006.doc]

***Acyrthosiphon pisum***

***The International Aphid Genomics Consortium***

**Table S6.** Distribution of reactions in the AcypiCyc database across the 6 top-level categories identified by the Enzyme Commission (EC). Included in this table are all reactions in the AcypiCyc database which have been assigned either full or partial EC numbers.

| EC Category | Number of reactions^1^ | | |
| --- | --- | --- | --- |
|  | *A. pisum* | *B. aphidicola* APS*^2^* | *D. melanogaster* |
| 1 -- Oxidoreductases | 267 (26%) | 62 (18%) | 220 (24%) |
| 2 -- Transferases | 344 (33%) | 134 (40%) | 286 (31%) |
| 3 -- Hydrolases | 264 (25%) | 45 (13%) | 240 (26%) |
| 4 -- Lyases | 68 (7%) | 33 (10%) | 67 (7%) |
| 5 -- Isomerases | 30 (3%) | 19 (6%) | 29 (3%) |
| 6 -- Ligases | 70 (7%) | 44 (13%) | 67 (7%) |
| Total | 1043 | 337 | 909 |

**^1^** Reactions with full or partial EC number are shown with % of total in parentheses

^2^ Data from SymbioCyc: http://pbil.univ-lyon1.fr/software/symbiocyc/
